# Identification of key molecular biomarkers involved in reactive and neurodegenerative processes present in inherited congenital hydrocephalus

**DOI:** 10.1186/s12987-021-00263-2

**Published:** 2021-07-02

**Authors:** Betsaida Ojeda-Pérez, José A. Campos-Sandoval, María García-Bonilla, Casimiro Cárdenas-García, Patricia Páez-González, Antonio J. Jiménez

**Affiliations:** 1 Department of Cell Biology, Genetics, and Physiology, Facultad de Ciencias, Universidad de Málaga, Campus de Teatinos, 29071 Malaga, Spain; 2 Instituto de Investigación Biomédica de Málaga (IBIMA), Malaga, Spain; 3 Servicios Centrales de Apoyo a la Investigación (SCAI), Universidad de Malaga, Malaga, Spain

**Keywords:** Biomarkers, Hereditary hydrocephalus, Energy dispersive X-ray spectroscopy (EDS), Lipids, Ultrahigh-performance liquid chromatography-high-resolution mass spectrometry (UHPLC–HRMS), Matrix-assisted laser desorption ionization mass spectrometry imaging (MALDI-MSI), NG2 antigen, Oligodendrocyte progenitor cells, Proteomic

## Abstract

**Background:**

Periventricular extracellular oedema, myelin damage, inflammation, and glial reactions are common neuropathological events that occur in the brain in congenital hydrocephalus. The periventricular white matter is the most affected region. The present study aimed to identify altered molecular and cellular biomarkers in the neocortex that can function as potential therapeutic targets to both treat and evaluate recovery from these neurodegenerative conditions. The hyh mouse model of hereditary hydrocephalus was used for this purpose.

**Methods:**

The hyh mouse model of hereditary hydrocephalus (hydrocephalus with hop gait) and control littermates without hydrocephalus were used in the present work. In tissue sections, the ionic content was investigated using energy dispersive X-ray spectroscopy scanning electron microscopy (EDS-SEM). For the lipid analysis, matrix-assisted laser desorption ionization mass spectrometry imaging (MALDI-MSI) was performed in frozen sections. The expression of proteins in the cerebral white matter was analysed by mass spectrometry. The oligodendrocyte progenitor cells (OPCs) were studied with immunofluorescence in cerebral sections and whole-mount preparations of the ventricle walls.

**Results:**

High sodium and chloride concentrations were found indicating oedema conditions in both the periventricular white matter and extending towards the grey matter. Lipid analysis revealed lower levels of two phosphatidylinositol molecular species in the grey matter, indicating that neural functions were altered in the hydrocephalic mice. In addition, the expression of proteins in the cerebral white matter revealed evident deregulation of the processes of oligodendrocyte differentiation and myelination. Because of the changes in oligodendrocyte differentiation in the white matter, OPCs were also studied. In hydrocephalic mice, OPCs were found to be reactive, overexpressing the NG2 antigen but not giving rise to an increase in mature oligodendrocytes. The higher levels of the NG2 antigen, diacylglycerophosphoserine and possibly transthyretin in the cerebrum of hydrocephalic hyh mice could indicate cell reactions that may have been triggered by inflammation, neurocytotoxic conditions, and ischaemia.

**Conclusion:**

Our results identify possible biomarkers of hydrocephalus in the cerebral grey and white matter. In the white matter, OPCs could be reacting to acquire a neuroprotective role or as a delay in the oligodendrocyte maturation.

**Supplementary Information:**

The online version contains supplementary material available at 10.1186/s12987-021-00263-2.

## Introduction

In hereditary congenital obstructive hydrocephalus, the elevation of intracranial pressure and enlargement of the cerebral ventricles induce injury in the brain parenchyma [1]. Consequently, there is a gradual stretching and destruction of periventricular white matter axons and a delay in their myelination [2]. Then, as in other pathological events, such as stroke or brain injury, axonal damage is considered the main pathological change [3–5]. The external capsule and corpus callosum have been described as the most susceptible white matter structures to suffer damage in hydrocephalus [4, 6–9]. Additionally, in congenital hydrocephalus, astrocyte and microglial cell activation is a common pathological event that can affect the cerebral white matter [9–17]. Activated microglia has been implicated in the destruction of the white matter in the early phases of hydrocephalus [17, 18]. Other harmful conditions affecting the periventricular walls in congenital hydrocephalus need to be considered such as the presence of periventricular oedema, which is reflected by the levels of osmolytes [19], and high levels of the proinflammatory cytokine TNFα and neuroexcitotoxic glutamate [19, 20].

In the present study, we looked for biomarkers of molecular and cellular responses under detrimental conditions that may also function as potential therapeutic targets. The hyh mouse model of hereditary congenital hydrocephalus [21] was used for this purpose. The origin and histopathology of hydrocephalus in the hyh mouse share similarities with that of hydrocephalus cases in humans [20, 22–24]. The hyh mouse presents a mutation in the *Napa* gene, which encodes N-ethylmaleimide-sensitive factor attachment protein alpha (α-Snap), which affects membrane trafficking and possibly cell adhesion in the neuroepithelium during development [24, 25]. As a consequence, the hyh mouse develops obstructive hydrocephalus with severe damage to the neocortex [17, 26–28].

The present study was focused on key pathophysiological events in hydrocephalus. First, the ionic environment was examined through an analysis of the elemental spectrum using scanning electron microscopy by energy dispersive X-ray spectroscopy (SEM–EDS). The presence of oedema conditions in the cerebrum, both in the periventricular white matter and deeper in the grey matter, was revealed. Additionally, the expression of lipids and proteins was investigated by matrix-assisted laser desorption ionization mass spectrometry imaging (MALDI-MSI) and ultrahigh-performance liquid chromatography-high-resolution mass spectrometry (UHPLC–HRMS), respectively. The results suggested a combination of neurodegenerative processes. Analysis of proteins in the white matter revealed deregulation of oligodendrocyte differentiation and myelination. For this reason, oligodendrocyte progenitor cells (OPCs) were also studied, and they were found to be reactive, but they did not appear to give rise to a higher density of mature oligodendrocytes. Our results in the neurodegenerative conditions studied in the brain parenchyma: i) suggest a delayed OPC differentiation and ii) show overexpression of neuron/glial NG2 antigen in the reactive OPCs that could play a neuroprotective role [29].

## Material and methods

### Experimental animals

Twenty-day-old mutant hydrocephalic hyh mice (hydrocephalus with hop gait, B6C3Fe- a/a-hyh/J strain) were used as a model of congenital obstructive hydrocephalus, and their non-hydrocephalic wild-type (wt) littermates were used as controls. The hyh mice were originally obtained from The Jackson Laboratory (Bar Harbor, ME, USA). They were bred by the Animal Experimentation Service of the University of Malaga on a 12:12 h light/dark cycle at 22 °C and provided standard food and water ad libitum. Wt and mutant hyh mice with severe hydrocephalus were identified by clinical inspection and genotyping [30]. The experimental procedures were approved by the Institutional Animal Care and Use Committee of the University of Malaga (CEUMA). Thus, the experiments were designed and the animals were housed, handled, cared for and processed in accordance with European and Spanish laws (RD53/2013 and 2010/63UE) and following the ARRIVE guidelines [31]. For EDS-SEM, MALDI-MSI, and UHPLC–HRMS, the animals were sacrificed by cervical dislocation. Then, the brains were quickly dissected under cold conditions, frozen in dry ice, and stored at − 80 °C. The procedure between dissection and freezing took less than five minutes. The mice were also anaesthetized with Dolethal (sodium pentobarbital; Vétoquinol, Lure, France; intraperitoneal administration, 0.2 mg/g body weight) and processed as described below to obtain brain sections or whole-mount preparations for immunofluorescence or haematoxylin–eosin staining.

### Scanning electron microscopy with analysis of the elemental spectrum

Frontal Sections (40 µm-thick) of the frozen brains of mice (wt, n = 3; hyh n = 3) were obtained with a cryostat (Leica CM1950, Nussloch, Germany) and mounted on slides. Then, the sections, corresponding in wt mice to the levels of coordinates between Bregma 1.1 mm and 0.14 mm according to the Paxinos´ atlas [32], were dried in an oven at 37 °C for 18 h, mounted on aluminium stubs, and sputtered with gold. Equivalent levels were analysed in the hyh mice. Although morphological artefacts are possible with frozen and drying procedures, the use of fixers or any wash that could change the concentration of ions was avoided. Images were obtained using a JEOL JSM-6490LV scanning electron microscope (SEM; Akishima, Tokyo, Japan) equipped with an EDS (energy dispersive X-ray analyser system) operating under high vacuum at 15 kV and a working distance of 10 mm. The EDS acquired an elemental spectrum, and then a SEM image showing the analysis area was obtained. Atomic percentages were obtained for the following selected elements: carbon (C), calcium (Ca), chloride (Cl), potassium (K), nitrogen (N), sodium (Na), magnesium (Mg), oxygen (O), phosphorus (P), and sulphur (S). Three to four areas of the white and grey matter in each image were analysed for a total of 2–3 sections.

### MALDI-MSI of lipids

For matrix-assisted laser desorption ionization mass spectrometry imaging (MALDI-MSI), the frozen brains of mice (wt, n = 3; hyh n = 3) were sectioned with a cryostat. The sections (10 µm-thick; frontal orientation; for wt mice levels at the coordinates between Bregma 1.1 mm and − 2.46 mm according to the Paxinos´ atlas [32].) were thaw-mounted on conductive ITO (indium tin oxide)-coated glass slides (Bruker Daltonics, Bremen, Germany). Then, the slides were dried inside a vacuum desiccator for 20 min and stored at − 80 °C until use. MALDI-MSI experiments were performed with an UltrafleXtreme MALDI TOF/TOF mass spectrometer equipped with a 2 kHz SmartBeam II Nd:YAG/355 nm (Bruker Daltonics) ionization source. Before analysis, the sample slides were placed inside a desiccator for 15 min. Matrix α-cyano-4-hydroxycinnamic acid (CHCA) was prepared at a concentration of 7 mg/mL in 60:40:0.2 (v:v:v) ACN:H_2_0:TFA and sprayed over the tissue samples using the SunCollect device (SunChrom, Friedrichsdorf, Germany). The spray settings were as follows: spray-head velocity, 850 mm/min; height, 1 mm; line distance, 2 mm; and flow rate, 60 μL/min. The total applied matrix was 2.9 μg/mm^2^.

Spectra were acquired in the m/z 600–1000 range in reflector negative ion mode. Prior to data acquisition, internal calibration was performed using the following signals of known lipid species: m/z 673.4814 (phosphatidic acid 34:1, [PA 34:1-H]^−^); m/z 715.4556 (phosphatidylglycerol 32:3, [PG 32:3-H]^−^), 747.5182 (phosphatidylglycerol 34:1, [PG 34:1-H]^−^), m/z 806.4978 (phosphatidylserine 38:6, [PS 38:6-H]^−^), m/z 834.5291 (phosphatidylserine 40:6, [PS 40:6-H]^−^), m/z 885.5499 (phosphatidylinositol 38:4, [PI 38:4-H]^−^), and m/z 906.6346 (sulfatide C24(OH), [ST 24(OH)-H]^−^). In total, 500 consecutive shots were irradiated per pixel at 5 different positions in random walk mode with the following parameters: laser energy, 45%; laser focus set, “medium”; ion source 1/2 voltage, 20 kV/17.8 kV; lens voltage, 7 kV; and pulsed ion extraction, 130 ns. The spatial resolution was 80 μm.

The spectral intensities were normalized to the total ion count, and ion images were generated with FlexImaging 4.0 software (Bruker Daltonics). A comparison of profiling spectra between sections from wt and hyh mice was performed with SCiLS Lab 2014b software.

To identify lipid species, MALDI-LIFT (MS/MS) of manually selected monoisotopic peaks was directly performed on tissue with the following settings: ion source 1/2 voltage, 7.5 kV/6.8 kV; lens voltage, 3.5 kV; LIFT voltage 1/2, 19 kV/4.2 kV; and precursor ion selector (PCIS) window range, 4 Da. Fragmentation data were compared with the Lipid MAPS database (www.lipidmaps.org).

### UHPLC–HRMS analysis of protein expression

For ultra-high-performance liquid chromatography-high-resolution mass spectrometry (UHPLC-HRMS), white matter was dissected from wt (n = 4) and hyh (n = 5) mice. Immediately after sacrificing by cervical dislocation, the white matter was dissected out under cold conditions, frozen on dry ice, and stored at -80 °C until further processing. The complete procedure took off between 2–5 min. For white matter extraction, lateral ventricles were opened to expose their surfaces. Then, the white matter, according to its microscopic appearance as shown in Additional file 1, was dissected out with a curved micro scissor for about 500 µm in deep. Proteins from the samples were purified by a modified trichloroacetic acid protein precipitation procedure (Clean-Up Kit; GE Healthcare, München, Germany), and gel-assisted proteolysis was carried out using a DigestPro MSi instrument (INTAVIS Bioanalytical Instruments AG, Cologne, Germany). Briefly, the protein solution was entrapped in a polyacrylamide gel matrix before reduction with dithiothreitol and cysteine residue carbamidomethylation with iodoacetamide. Then, the proteins were digested by trypsin (Promega, Madison, WI, USA), and peptides were extracted from the gel with an acetonitrile/formic acid solution. After extraction, the peptides were purified and concentrated using a C18 ZipTip (Merck Millipore, Darmstadt, Germany) according to the manufacturer’s instructions.

Samples were injected into an Easy nLC 1200 UHPLC system coupled to a Q Exactive™ HF-X Hybrid Quadrupole-Orbitrap mass spectrometer (Thermo Fisher, Waltham, MA, USA). Data were acquired using Tune 2.9 and Xcalibur 4.1.31.9 (Thermo Fisher). Peptides from the samples were automatically loaded into a trap column (Acclaim PepMap 100, 75 µm × 2 cm, C18, 3 µm, 100 A, Thermo Fisher) and eluted onto a 50 cm analytical column (PepMap RSLC C18, 2 µm, 100 A, 75 µm × 50 cm, Thermo Fisher). The binary gradient mobile phase consisted of 0.1% formic acid in water (solvent A) and 0.1% formic acid in 80% acetonitrile (solvent B). Peptides were eluted from the analytical column with a 120 min gradient from 2 to 20% solvent B, followed by a 30 min gradient from 20 to 35% solvent B and finally in 95% solvent B for 15 min before re-equilibration in 2% solvent B at a constant flow rate of 300 nL/min.

Data acquisition was performed in electrospray ionization positive mode. MS1 scans were acquired from m/z 300–1750 at a resolution of 120,000. Using a data-dependent acquisition method, the 20 most intense precursor ions with + 2 to + 5 charges were isolated within a 1.2 m/z window and fragmented to obtain the corresponding MS/MS spectra. The fragment ions were generated in a higher-energy collisional dissociation cell with a fixed first mass at 110 m/z and detected by the Orbitrap mass analyser at a resolution of 30,000.

The raw data were analysed using Proteome DiscovererTM 2.2 (Thermo Fisher). For the identification of the MS2 spectra, Sequest HT was utilized as the search engine, and the Swiss-Prot part of UniProt for *Mus musculus* was utilized as the database. Protein assignments were validated using the Percolator algorithm [33] by imposing a strict cut-off of a 1% false discovery rate (FDR).

Label-free quantitation was implemented using the Minora feature of Proteome DiscovererTM 2.2. Protein abundance ratios were calculated based on unique peptides as the median of all possible pairwise ratios calculated between replicates of all connected peptides.

The PANTHER Classification System (v.14.1) [34] and DAVID Bioinformatics Resources (6.8) [35] were used to identify the main biological processes related to the overexpressed/underexpressed proteins. Functional pathways were analysed using the Kyoto Encyclopedia of Genes and Genomes (KEGG) database [36].

### Immunofluorescence

**Fig. 1:**
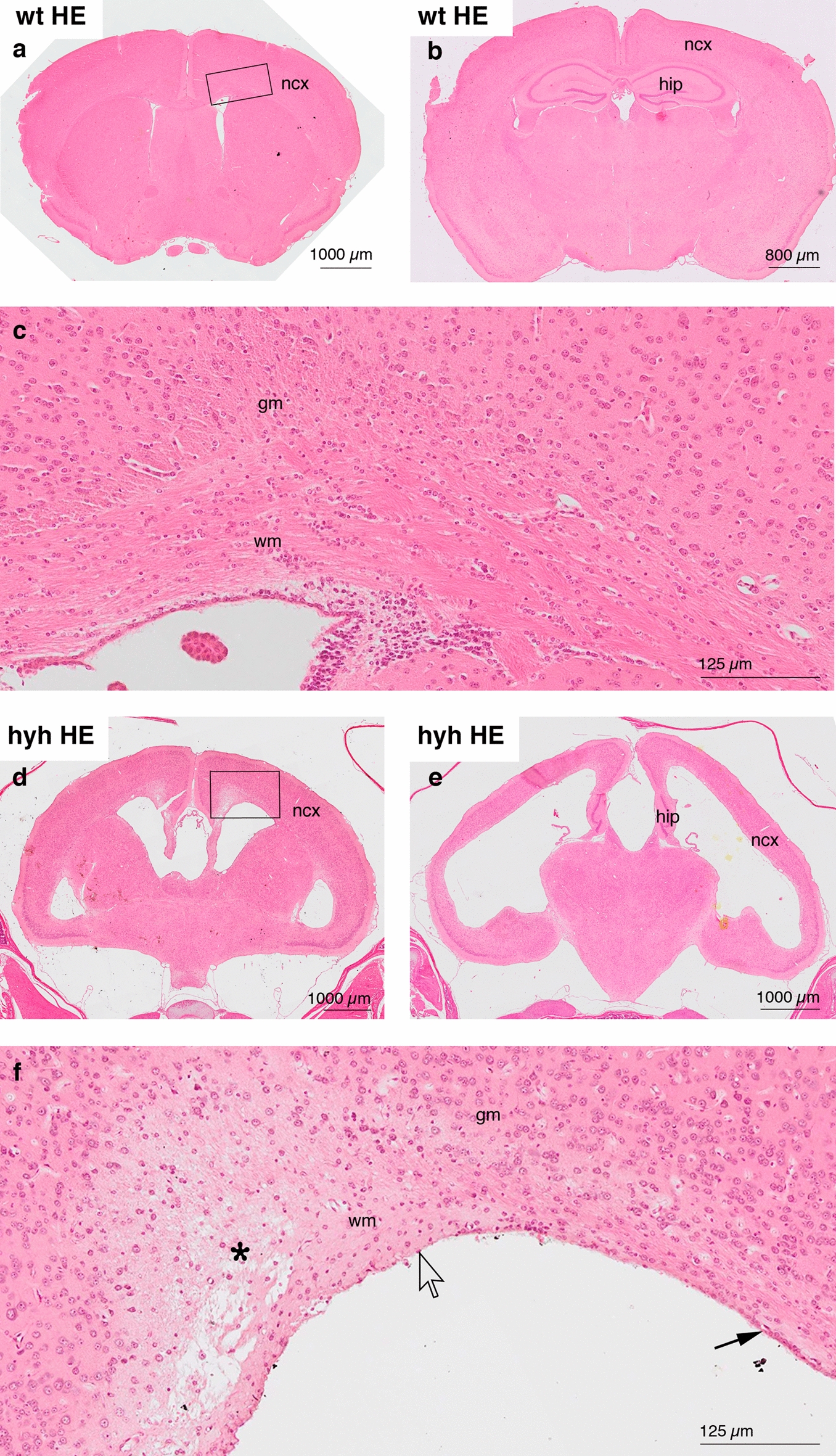
Histopathology of the brain in hyh mice. Haematoxylin–eosin staining (HE).** a–c** Frontal paraffin sections of the neocortex (*ncx*) of a wt mouse at two different levels. The area framed in **a**, which shows the grey (*gm)* and white (*wm*) matter, is detailed in **c**. **d–f** Frontal paraffin sections of a hyh mouse at the levels of the anterior commissure and the rostral hippocampus (*hip*). The area framed in **d**, which shows the affected and oedematous (*asterisk*) white matter, is detailed in **f**. Most of the ventricle surface (*open arrow*) of the hyh mouse lacks ependyma (*black arrow*)

Hyh mice and wt mice were used for four different experimental approaches: (i) the use of frontal brain sections (wt n = 3; hyh n = 3) and whole-mount preparations (wt, n = 2; hyh n = 2) for co-labelling of NG2 and other cell markers; (ii) the use of frontal brain sections to quantify NG2 immunoreactivity and the density of NG2-positive cells (wt, n = 4; hyh, n = 4); (iii) the use of frontal brain sections to quantify the densities of NG2- and Olig2-positive cells (wt, n = 6; hyh, n = 6); and (iv) the use of frontal brain sections (wt, n = 4; hyh, n = 5) to quantify the proliferation of NG2 cells 24 h after a single intraperitoneal dose of bromodeoxyuridine (BrdU, 100 mg/kg). Anaesthetized mice were transcardially perfused with 4% paraformaldehyde diluted in 0.1 M phosphate buffer, pH 7.4. The fixed brains were removed, postfixed in the same solution for 24 h at 4 °C, and then cryoprotected in 30% sucrose. Frozen frontal brain sections (60 μm thick) at the levels shown in Fig. 1a, d (inserts). were obtained for immunostaining. The levels used for labelling were comprised approximately between the coordinates Bregma 1.1 mm and 0.14 mm, for wt mice, according to Paxinos´ atlas [32]. Equivalent levels for hyh mice were used. For BrdU immunostaining, sections were pre-treated with HCl (0.2 N) for 10 min at 37 °C. In the case of whole-mount preparations, the neocortical wall was dissected after postfixation.

Frozen sections and whole-mounts were immunostained according to free-floating procedures. Antibodies against the following antigens were used: NG2 (Merck Millipore; AB5320, rabbit polyclonal, 1:400 and 1:1000 dilutions); GFAP (glial fibrillary acidic protein; Sigma, St Louis, MO, USA; G3893, mouse monoclonal, 1:1000 dilution); Olig2 (R&D Systems, Minneapolis, MN, USA; AF2418, goat polyclonal, 1:1000 dilution); NeuN (Merck Millipore; MAB377, mouse monoclonal, 1:500 dilution); TTR (Invitrogen, Carlsbad, CA, USA; PA5-80197, rabbit polyclonal, 1:1000 dilution), and BrdU (Developmental Studies Hybridoma Bank, Iowa City, IA, USA; G3G4 clone, mouse monoclonal, 1:1000 dilution). Primary antibody incubations were performed for 18 h at 22 °C or for 72 h at 4 °C. The secondary antibodies that were used were conjugated to Alexa Fluor 488 or Alexa Fluor 568 (Thermo Fisher) or biotinylated (Sigma). Incubations with secondary antibodies and streptavidin conjugated to Alexa Fluor 488 and 568 (Thermo Fisher) were performed for 1 h at 22 °C. The antibodies used for immunostaining were diluted in PBS comprising 0.05% Triton X-100 (Sigma) and 0.01% sodium azide. Nuclear staining was performed with 4',6-diamidino-2-phenylindole (DAPI, Molecular Probes, Eugene, OR, USA). Immunofluorescence images were obtained with Leica SP5 and SP8 laser confocal microscopes (Leica, Wetzlar, Germany) using a hybrid sensor (HyD). For each experiment, images were acquired with the same settings. For negative controls, the primary antibodies were omitted.

### Haematoxylin–eosin staining

Anaesthetized mice (wt, n = 2; hyh n = 2) were transcardially perfused with Bouin´s fixative solution. Then, the brains were postfixed for 72 h and embedded in paraffin to obtain serial frontal sections (10 μm thick). Deparaffinated sections were stained with haematoxylin–eosin. The stained sections were scanned using a VS120 microscope (Olympus, Tokyo, Japan) with a UPLSAPO20x/0.75 objective.

### Data analyses and statistics

Quantification of the confocal images was carried out on the original micrographs. The densities (cells/area) of NG2 + cells and BrdU + cells in the frontal cortex white matter were calculated in 4 fields per section (10 µm thickness) for each animal. Analyses were blinded by using different researchers and by masking the samples. The density of immunoreactivity was quantified using ImageJ (https://imagej.net/Welcome). Statistical analyses were performed using KaleidaGraph (Synergy Software, Reading, PA, USA). All values are reported in the figures as the mean with 95% confidence. The Wilcoxon-Mann–Whitney test and Student’s t-test were applied to test the hypothesis in situations requiring non-parametric and parametric analyses, respectively. When the *F* probability obtained by Student’s t-test was < 0.05, the variance was considered unequal. *P* < 0.05 was considered statistically significant for both tests. For PANTHER and mass spectrometry analyses, *p*-values are indicated in the tables. In the spectrometry analysis, abundance ratio *p*-values were calculated by ANOVA based on the background population of peptides and proteins. This method uses the background population of ratios for all peptides and proteins to determine whether any given a single peptide or protein is significantly changing relative to that background. For PANTHER analyses, binomial tests with Bonferroni correction for multiple testing with P < 0.05 were used.

## Results

The hyh mouse presented severe obstructive hydrocephalus at 20 days of age, and it was observed that the periventricular white matter in particular was seriously affected and exhibited an oedematous appearance (Fig. 1). In contrast, no effect on neurons in the grey matter was detected by general histological staining (Fig. 1). For this reason, the focus of the subsequent investigation was on the extent of the oedema conditions.

### Analysis of elements using energy dispersive X-ray spectroscopy scanning electron microscopy

**Fig. 2:**
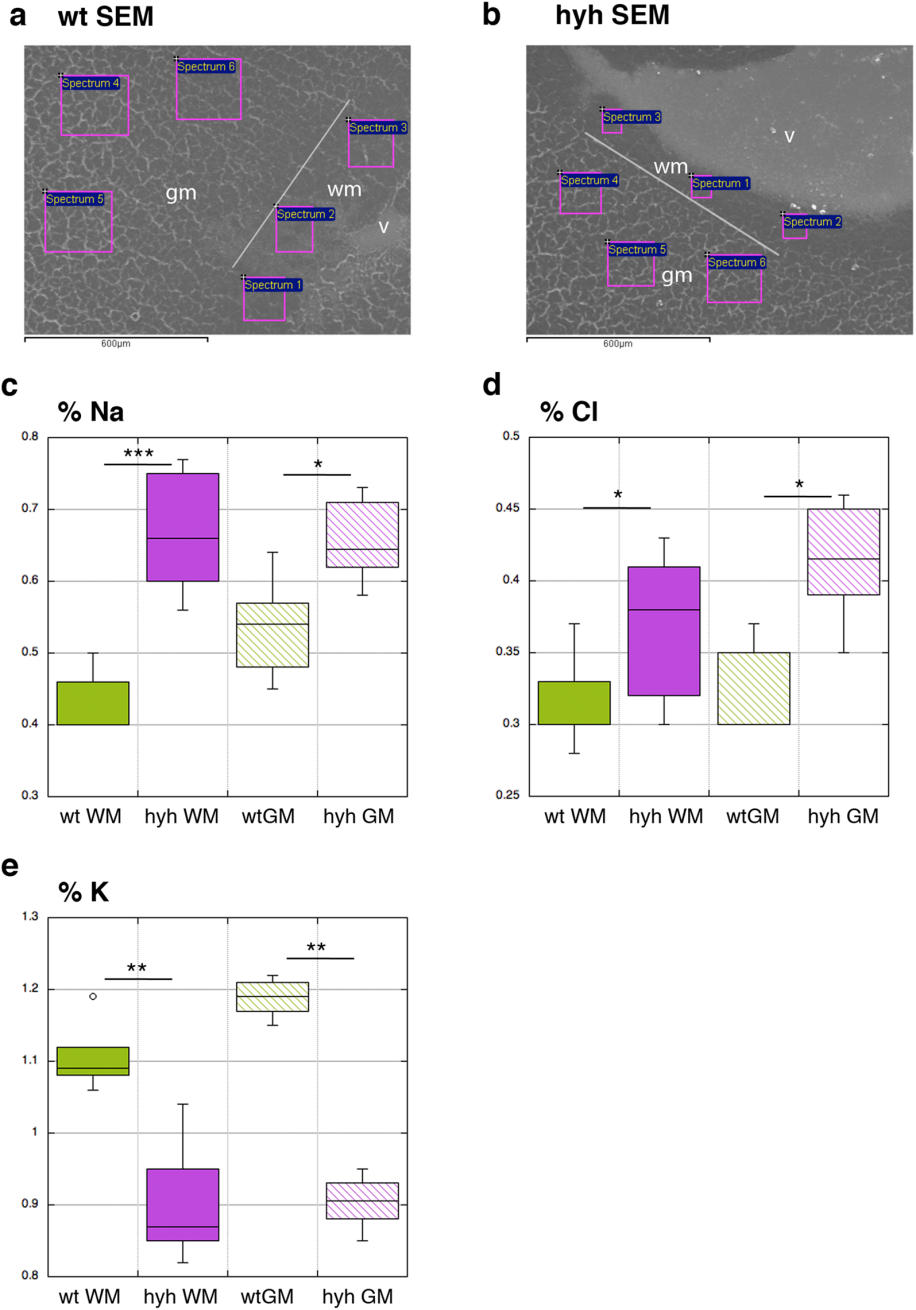
Analysis of the elemental spectra using EDS-SEM. **a**, **b** Representative SEM images showing areas in wt (**a**) and hyh (**b**) mice selected to obtain spectra for quantification. The border between the grey (*gm*) and white (*wm*) matter is shown (*white lines* in **a** and **b**). **c–e** Atomic percentages of sodium (**c**, *Na*), chloride (**d**, *Cl*), and potassium (**e**, *K*) in the white and grey matter of wt (n = 3) and hyh mice (n = 3) indicating the presence of oedema. *v* lateral ventricle. *p < 0.05, **p < 0.005; ***p < 0.0005; Student’s t-test

To determine whether oedema was present, the relative atomic percentages of sodium, potassium, and chloride were analysed by EDS-SEM. These ions were selected for the analysis because chloride and water transport has been reported to occur following sodium transport to maintain electrical and osmotic neutrality to form ionic oedema [37]. Spectra were obtained for these elements in the white and grey matter of hyh mice and compared with those in the white and grey matter of wt mice. In the white and adjacent grey matter of hydrocephalic mice, increased concentrations of sodium and chloride and an opposite trend in potassium levels were detected, thus revealing the presence of oedema (Fig. 2). Other elements, including calcium, as is shown in the Additional file 2, did not show differences between wt and hyh mice.

### Analysis of lipids in the cerebrum

**Fig. 3:**
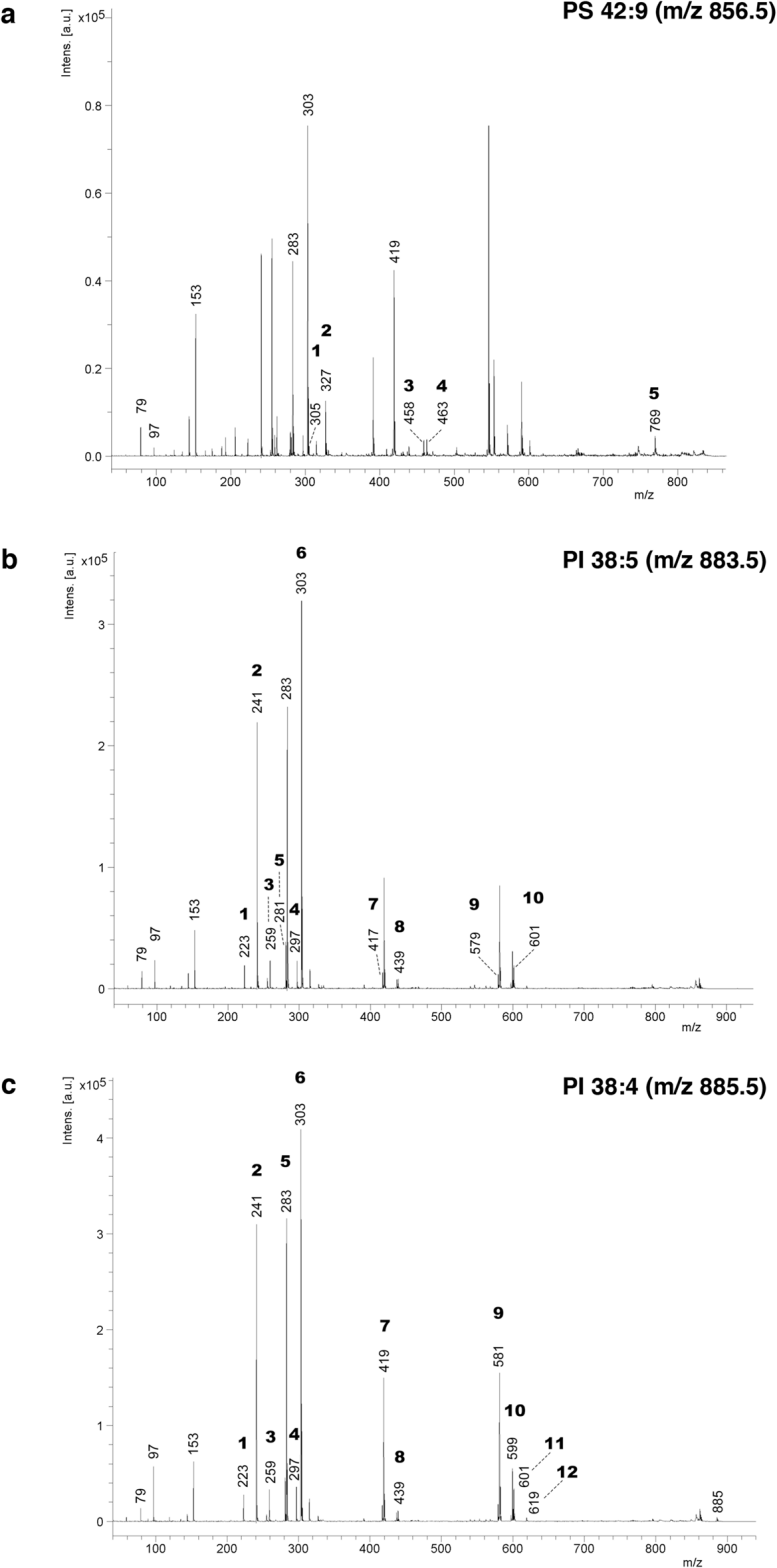
MS/MS spectra of lipids acquired in negative mode showing differences between wt and hyh mice. **a** PS (42:9) (m/z 856.5) spectrum. Fragments 1 and 2 correspond to the carboxylated 20:3 and 22:6 chains, respectively. Ions 3 and 4 correspond to neutral loss of 22:6 as a ketene and serine and to neutral loss of 20:3 as RCOOH and serine, respectively. Fragment 5 represents the loss of serine from [M-H]-. The spectrum also shows the fragmentation pattern of the more abundant PI (36:4) (m/z 857.5). **b** PI (38:5) (m/z 883.5) spectrum. Fragments shared with the more abundant PI (38:4): 1, 2, 3, 4 (different forms of inositol phosphate ions) and 6 (carboxylated 20:4 chain). Ion 5 is the carboxylated 18:1 chain. Ion 7 represents the neutral loss of the 20:4 RCOOH group and inositol from [M-H]-. Ion 8 represents the neutral loss of the 18:1 chain and inositol and/or the neutral loss of 18:0 and inositol from PI (38:4). Fragments 9 and 10 represent the neutral loss of 20:4 and 18:1 as RCOOH groups, respectively. **c** PI (38:4) (m/z 885.5) spectrum. Fragments 1, 2, 3, and 4 correspond to inositol phosphate (IP)–2H_2_O, IP –H_2_O, IP, and IP + glycerol backbone ions, respectively. Ions 5 and 6 are the carboxylated 18:0 and 20:4 chains, respectively. Fragments 7 and 8 represent the neutral loss of 20:4 and 18:0 RCOOH groups and inositol from [M-H]-, respectively. Ions 9 to 12 correspond to the neutral loss of 20:4 and 18:0 either as RCOOH or as ketenes, respectively. The m/z 885 peak is the precursor ion [M-H]-

**Fig. 4:**
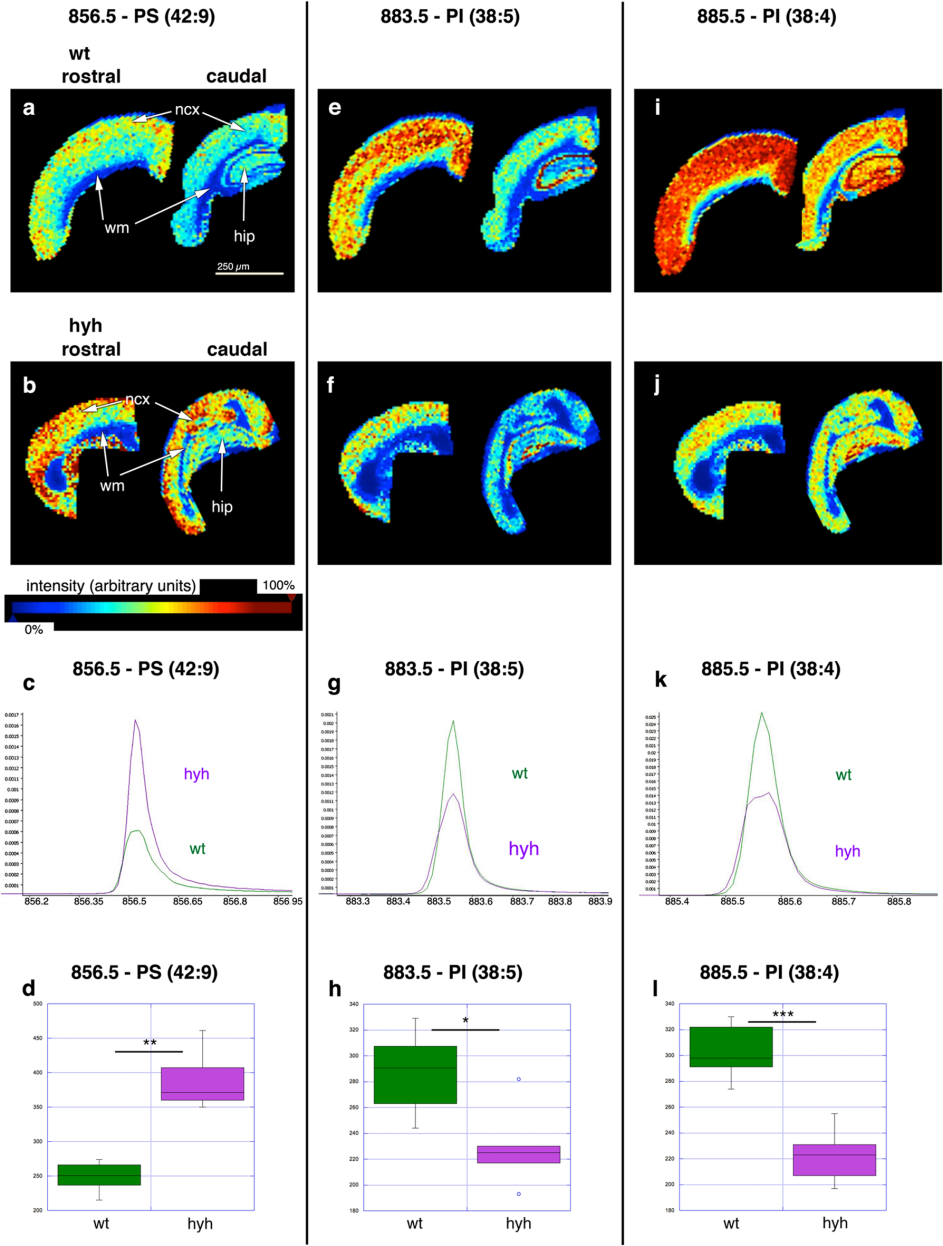
Analysis of lipids by MALDI-MSI showing differential expression between wt and hydrocephalic hyh mice. Ion images of diacylglycerophosphoserine PS (42:9), diacylglycerophosphoinositol PI (38:5), and PI (38:4) in neocortical (*ncx*) frontal sections of wt (**a, e, i**) and hyh (**b, f, j**) mice obtained in negative ion mode at a spatial resolution of 80 μm. Images of frontal sections in pairs: left image: rostral neocortex (anterior commissure level); right image: caudal neocortex adjacent to hippocampus (*hip*). The white (*wm*) and grey (*gm*) matter are labelled. The *heat bar* represents the relative intensity, indicating the abundance of each ion. The averaged monoisotopic peak for each lipid (**c, g, k**) and the quantification of these peaks are shown (**d, h, l**). *p < 0.05, **p < 0.005, ***p < 0.0005; Student’s t-test (wt, n = 3; hyh n = 3)

Considering that oedema conditions extended towards the grey matter and that axons in the white matter could be affected, experiments were designed to identify lipid biomarkers of neural damage and cell reactions in both regions, the white and grey matter. MALDI-MSI was used for this purpose. This method has been proven useful for analysis of the distribution of lipids in relation to neurodegenerative processes [38, 39]. Spectra acquired in reflector negative ion mode showed consistent differences in lipid content in the grey matter of wt and hyh mice but not in the white matter. In the negative ion mode, there was only a reliable detection of PI and PS molecular species. The possibility of differences in other lipids in the white matter cannot be excluded because they would need detection in the positive ion mode. Notably, in the spectra obtained in negative ion mode, three lipid species could be identified that showed differences in expression between hyh and wt mice. These lipids were identified after fragmentation by MALDI-LIFT (MS/MS) (Fig. 3). A peak with m/z 856.5 Da was increased in the hyh mouse neocortex compared with the wt mouse neocortex (Fig. 4). This lipid, which was therefore found at a higher concentration in the grey matter in hyh mice than in wt mice, was identified as the diacylglycerophosphoserine molecular species PS (42:9), which contains 20:3 and 22:6 fatty-acyl chains (Fig. 3). Two other peaks showed the opposite trend, and their levels were higher in the cerebral grey matter in wt mice than in hyh mice (Fig. 4). These peaks, with m/z 883.5 and 885.5 Da, corresponded to the diacylglycerophosphoinositol (PI, phosphatidylinositol) molecular species PI (38:5) (which has 18:1 and 20:4 fatty acyl chains) and PI (38:4) (which has 18:0 and 20:4 fatty acyl chains), respectively (Fig. 3). Both PI molecular species appeared to be mainly expressed in the neurons, as they were strongly expressed in the granular layer of the hippocampus (Fig. 4e, i, f, j). This expression pattern was in contrast with the expression pattern of PS (42:9) (Fig. 4a, b).

### Analysis of proteins in the white matter

**Fig. 5:**
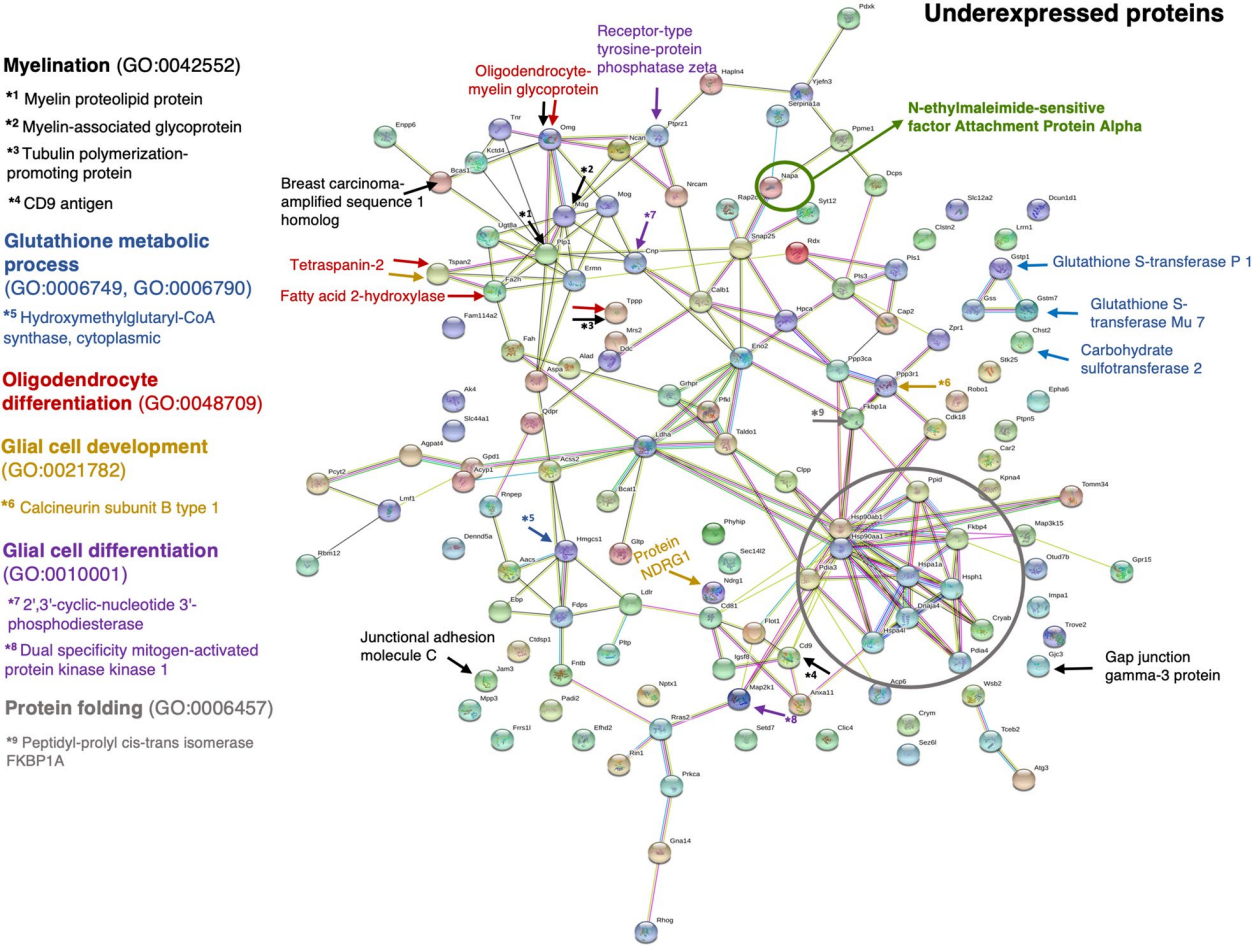
Analysis of underexpressed proteins in the white matter of hyh mice. Functional enrichment analysis of the protein–protein interaction network was performed with the search tool for the retrieval of interacting genes/proteins (STRING). Protein interactions implicated in the different biological processes presented in Tables 1, 2, 3, 4, 6, 7, 9, and 11 are shown. The network nodes are proteins, and the edges represent the predicted functional associations. All nodes are query underexpressed proteins in the white matter of hyh mice (n = 5) compared with wt mice (n = 4) identified by proteomic analysis. The filled nodes indicate proteins with known or predicted 3D structures, and the empty nodes represent proteins with unknown 3D structures. The edges are drawn according to the type of evidence used to predict the associations, with different coloured lines indicating the following 7 different types of evidence: fusion evidence (*red line*), neighbourhood evidence (*green line*), cooccurrence evidence (*blue line*), experimental evidence (*purple line*), textmining evidence (*yellow line*), database evidence (*light blue*), and coexpression evidence (*black line*). The GO processes in which the proteins are implicated are indicated by letters, arrows, or circles of colours (*black, blue, red, yellow, purple, and grey*). The decrease in the level of α-Snap (N-ethylmaeimide-sensitive factor attachment protein alpha), which is characteristic of hyh mice, is shown

**Fig. 6:**
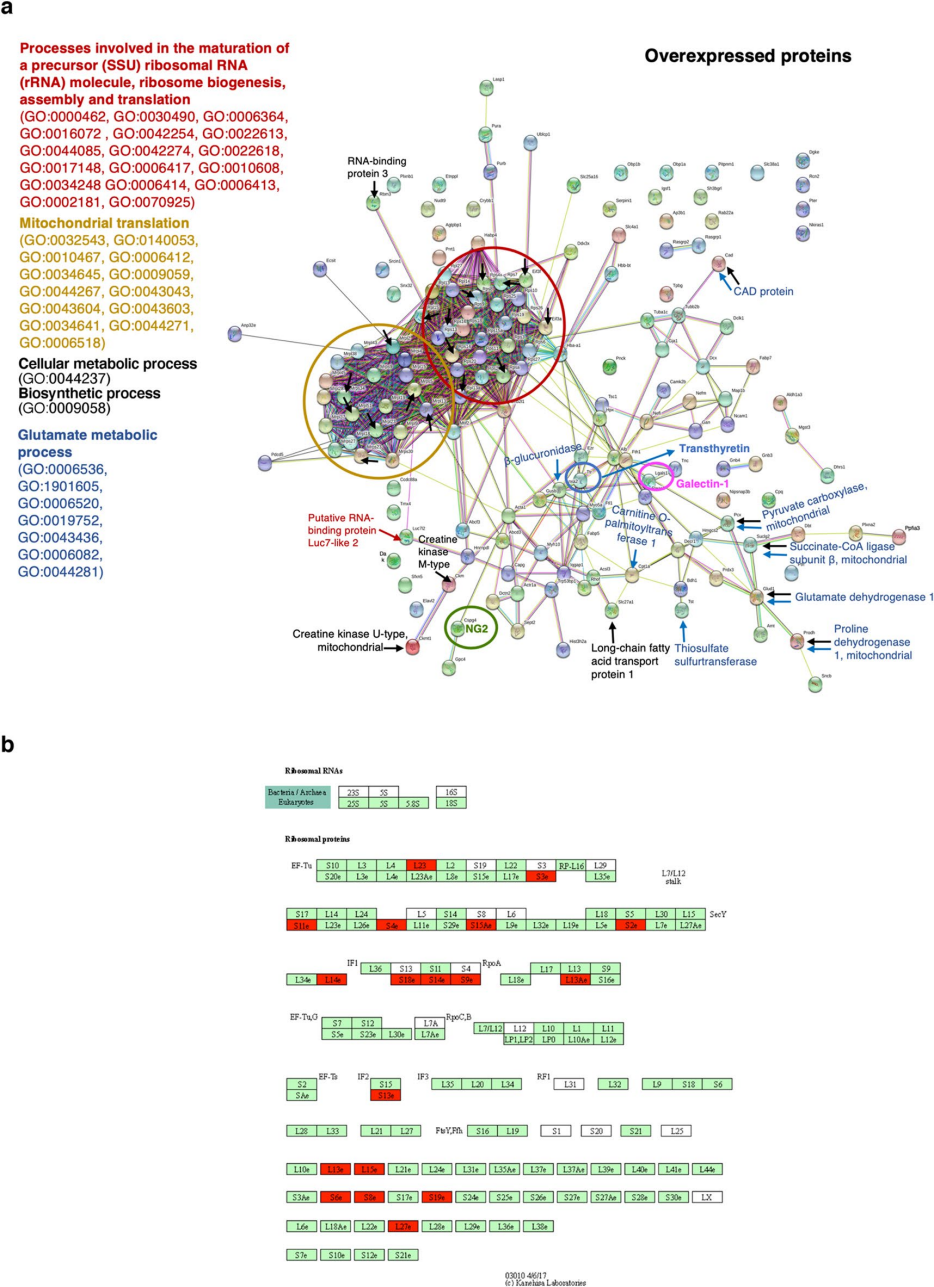
Analysis of overexpressed proteins in the white matter of hyh mice. **a** STRING analysis of overexpressed proteins in the white matter of hyh mice (n = 5) compared with wt mice (n = 4). Protein interactions implicated in the different biological processes presented in Tables 5, 8, 10, and 11 are shown. The network nodes are proteins, and the edges represent the predicted functional associations. All nodes are overexpressed proteins identified in the white matter of hyh mice by proteomic analysis. The filled nodes indicate proteins with known or predicted 3D structures, and the empty nodes represent proteins with an unknown 3D structure. The edges are drawn according to the type of evidence used to predict the associations, with different coloured lines indicating the following 7 different types of evidence: fusion evidence (*red line*), neighbourhood evidence (*green line*), cooccurrence evidence (*blue line*), experimental evidence (*purple line*), textmining evidence (*yellow line*), database evidence (*light blue*), and coexpression evidence (*black line*). The colours of the letters, arrows, and circles (*red, yellow, black, and blue*) indicate their respective GO processes. **b** KEGG pathway map for ribosomes from *Mus musculus* (pathway: mmu03010). Mouse ribosomal proteins are shown in *green*, and proteins overexpressed in hyh mice are shown in *red*. The image was obtained by a valid licence from the KEGG module within Proteome Discoverer software (Thermo Scientific)

Because white matter was observed to be the main affected region, an investigation to identify the molecular and cellular responses by UHPLC-HRMS was performed in this particular brain area. The grey matter was excluded from the extracts to be analysed to allow easier identification of the underexpressed and overexpressed proteins. The analysis showed underexpression of proteins related to oligodendrocyte development and differentiation, including myelination, in the white matter of hyh mice (Tables 1, 2, 3 and 4, Fig. 5). These proteins included myelin-associated glycoprotein, myelin proteolipid protein, and oligodendrocyte-myelin glycoprotein. This trend contrasted with the overexpression of proteins implicated in metabolism (Table 5, Fig. 6) and in the astrocyte reaction, as expected for hydrocephalus according to the literature, such as GFAP and vimentin (shown in the Additional file 3) and the NG2 antigen (Fig. 6), the latter present in OPCs. Analysis of the white matter in hyh mice also showed underexpression of proteins related to the antioxidant glutathione metabolism and protein folding (Tables 6 and 7, Fig. 5) but overexpression of proteins related to glutamate metabolism (Tables 8 and 9, Fig. 6).

**Table 1:** Underexpressed biological processes related to oligodendrocyte development

GO biological process category	Child category	Child category	Child category	Child category	Number of genes process	Number of genes underexpressed	Expected value	Fold enrichment	P-value
System development (GO:0048731)					4219	50	24.98	2.00	2.61E-03
	Nervous system development (GO:0007399)				2264	38	13.40	2.84	1.82E-05
		Neurogenesis (GO:0022008)			1771	31	10.48	2.96	3.26E-04
			Gliogenesis (GO:0042063)		235	11	1.39	7.91	1.70E-03
				Glial cell differentiation (GO:0010001)	185	11	1.10	10.04	1.57E-04
Anatomical structure development (GO:0048856)					5208	56	30.83	1.82	8.78E-03
	Glial cell development (GO:0021782)				113	8	0.67	11.96	4.08E-03
		Oligodendrocyte development (GO:0014003)			41	5	0.24	20.60	4.76E-02
Central nervous system development (GO:0007417)					835	20	4.94	4.05	1.07E-03
	Oligodendrocyte differentiation (GO:0048709)				68	8	0.40	19.87	8.76E-05

Identification of biological processes related to underexpressed proteins in the white matter of hydrocephalic hyh mice compared with normal (wt) mice. PANTHER GO biological process complete annotation data set by application of a binomial test with Bonferroni correction for multiple testing with P < 0.05 was used. Gene ontology (GO) biological process terms are indicated. The expected value is the number of expected genes in the list for the category based on the reference list. A fold enrichment indicates that the category was overrepresented in hyh mice compared to wt mice

**Table 2:** Underexpressed proteins implicated in the oligodendrocyte development

GO biological process class/child class	Gene name	Sum PEP score	Abundance ratio: (Sample) / (Control)	Abundance ratio P-value: (sample) / (control)
Oligodendrocyte development				
	Fatty acid 2-hydroxylase* **	4.50	0.436	0.001
	Myelin-associated glycoprotein* **	177	0.341	2.22E-14
	Myelin proteolipid protein* **	42.4	0.324	1.33E-15
	Oligodendrocyte-myelin glycoprotein* **	28.9	0.667	0.027
	Tubulin polymerization-promoting protein * **	84	0.674	0.009
Glial development*				
	Calcineurin subunit B type 1 **	62.58	0.72	0.047
	Protein NDRG1 **	35.04	0.666	0.043
	Tetraspanin-2 **	24.51	0.283	2.36E-11
Glial cell differentiation**				
	2',3'-cyclic-nucleotide 3'-phosphodiesterase	485.39	0.401	1.16E-10
	Dual specificity mitogen-activated protein kinase kinase 1	203.85	0.661	0.006
	Receptor-type tyrosine-protein phosphatase zeta	196.06	0.726	0.039

Underexpressed proteins in the white matter of hyh mice implicated in the processes of oligodendrocyte development. *; **, Proteins in the classes are included in their respective child classes (see Table 1). The sum PEP score corresponds to the score calculated based on the posterior error probability (PEP) values of the peptide spectrum matches (PSM). The PEP indicates the probability that an observed PSM is a random event. Sum PEP score is calculated as the negative logarithms of the PEP values of the connected PSM

**Table 3:** Underexpressed biological processes related to myelination and axon development

GO biological process	Child category	Child category	Number of genes process	Number of genes underexpressed	Expected value	Fold enrichment	P-value
Ensheathment of neurons (GO:0007272)			114	12	0.67	17.78	5.62E-08
	Axon ensheathment (GO:0008366)		114	12	0.67	17.78	5.62E-08
		Myelination (GO:0042552)	112	12	0.66	18.10	4.59E-08
Generation of neurons (GO:0048699)			1663	27	9.85	2.74	1.28E-02
	Neuron projection development (GO:0031175)		691	17	4.09	4.16	7.19E-03
		Axon development (GO:0061564)	373	15	2.21	6.79	6.83E-05

Identification of biological processes related to underexpressed proteins in the white matter of hydrocephalic hyh mice compared with normal (wt) mice. PANTHER GO biological process complete annotation data set by application of a binomial test with Bonferroni correction for multiple testing with P < 0.05 was used. Gene ontology (GO) biological process terms are indicated. The expected value is the number of expected genes in the list for the category based on the reference list. A fold enrichment indicates that the category was overrepresented in hyh mice compared to wt mice

**Table 4:** Underexpressed proteins implicated in myelination

Gene name	Sum PEP score	Abundance ratio: (sample) / (control)	Abundance ratio P-value: (sample) / (control)
Breast carcinoma-amplified sequence 1 homolog	79.326	0.51	5.41E-06
Calcineurin subunit B type 1	62.58	0.72	0.045
CD9 antigen	6.995	0.584	0.017
Gap junction gamma-3 protein	18.265	0.324	9.08E-08
Junctional adhesion molecule C	4.494	0.252	7.69E-11
Myelin-associated glycoprotein*	177	0.341	2.22E-14
Myelin proteolipid protein*	42.4	0.324	1.33E-15
Oligodendrocyte-myelin glycoprotein*	28.9	0.667	0.026
Protein NDRG1e	35.044	0.666	0.042
Tetraspanin-2	24.519	0.283	2.36E-11
Tubulin polymerization-promoting protein	84.096	0.674	0.009

Underexpressed proteins in the white matter of hyh mice implicated in myelination in the white matter of hyh mice. All the proteins are present in the class and child classes “myelination”, “axon ensheathment”, and “ensheathment of neurons” (see Table 3). *Proteins involved in oligodendrocyte development and differentiation. For meaning of sum PEP score see Table 2 legend

**Table 5:** Overexpressed biological processes related to metabolism

GO-slim biological process category	Child category	Child category	Number of genes process	Number of genes overexpressed	Expected value	Fold enrichment	P-value
Cellular metabolic process (GO:0044237)			4357	40	30.53	1.31	3.83E-02
	Organonitrogen compound metabolic process (GO:1901564)		2567	28	17.71	1.58	9.64E-03
	Ammonium ion metabolic process (GO:0097164)		36	2	0.25	7.03	2.68E-02
Biosynthetic process (GO:0009058)			2006	22	14.06	1.57	2.38E-02
	Organic substance biosynthetic process (GO:1901576)		1986	22	13.91	1.58	2.15E-02
		Organonitrogen compound biosynthetic process (GO:1901566)	589	18	4.13	4.36	2.09E-07
		Organophosphate biosynthetic process (GO:0090407)	167	4	1.17	3.42	3.07E-02
	Phospholipid biosynthetic process (GO:0008654)		50	2	0.35	5.71	4.86E-02

Identification of biological processes related to overexpressed proteins in the white matter of hydrocephalic hyh mice compared with normal (wt) mice. PANTHER GO Slim biological process annotation data set by application of a binomial test with Bonferroni correction for multiple testing with P < 0.05 was used. Gene ontology (GO) biological process terms are indicated. The expected value is the number of expected genes in the list for the category based on the reference list. A fold enrichment indicates that the category was overrepresented in hyh mice compared to wt mice

**Table 6:** Underexpressed biological processes related to glutathione metabolism

GO biological process	Child category	Number of genes process	Number of genes underexpressed	Expected value	Fold enrichment	P-value
Sulfur compound metabolic process (GO:0006790)		118	4	0.70	5.72	5.57E-03
	Glutathione metabolic process (GO:0006749)	35	2	0.21	9.64	1.87E-02

Identification of biological processes related to underexpressed proteins in the white matter of hydrocephalic hyh mice compared with normal (wt) mice. PANTHER GO biological process complete annotation data set by application of a binomial test with Bonferroni correction for multiple testing with P < 0.05 was used. Gene ontology (GO) biological process terms are indicated. The expected value is the number of expected genes in the list for the category based on the reference list. A fold enrichment indicates that the category was overrepresented in hyh mice compared to wt mice

**Table 7:** Underexpressed proteins implicated in glutathione metabolism

GO-slim biological process class/child class	Gene name	Sum PEP score	Abundance ratio: (sample) / (control)	Abundance ratio P-value: (sample) / (control)
Glutathione metabolic process				
	Glutathione S-transferase Mu 7*	66.1	0.667	0.027
	Glutathione S-transferase P 1*	106.96	0.727	0.039
Sulfur compound metabolic process*				
	Carbohydrate sulfotransferase 2	7.42	0.425	2.14E-5
	Hydroxymethyl-glutaryl-CoA synthase	119.39	0.688	0.014

Underexpressed proteins implied in glutathione metabolism in the white matter of hyh mice. *Proteins in the class are also included in the respective child class (see Table 6). For meaning of sum PEP score see Table 2 legend

**Table 8:** Overexpressed biological processes related to glutamate metabolism

GO-slim biological process Category	Child category	Child category	Child category	Child category	Child category	Child category	Number of genes process	Number of genes overexpressed	Expected value	Fold enrichment	P-value
Small molecule metabolic process (GO:0044281)							587	11	4.11	2.67	3.02E-03
	Organic acid metabolic process (GO:0006082)						355	7	2.49	2.81	1.31E-02
		Oxoacid metabolic process (GO:0043436)					342	7	2.40	2.92	1.08E-02
			Carboxylic acid metabolic process (GO:0019752)				331	7	2.32	3.02	9.19E-03
				Cellular amino acid metabolic process (GO:0006520)			123	4	0.86	4.64	1.14E-02
					Alpha-amino acid metabolic process (GO:1901605)		77	4	0.54	7.41	2.24E-03
						Glutamate metabolic process (GO:0006536)	4	2	0.3	71.36	3.83E-04

Identification of biological processes related to overexpressed proteins in the white matter of hydrocephalic hyh mice compared with normal (wt) mice. PANTHER GO Slim biological process annotation data set by application of a binomial test with Bonferroni correction for multiple testing with P < 0.05 was used. Gene ontology (GO) biological process terms are indicated. The expected value is the number of expected genes in the list for the category based on the reference list. A fold enrichment indicates that the category was overrepresented in hyh mice compared to wt mice

**Table 9:** Underexpressed proteins implicated in the glutamate metabolism

GO-slim biological process class/child class	Gene name	Sum PEP Score	Abundance ratio: (sample) / (control)	Abundance ratio P-value: (sample) / (control)
Glutamate metabolic process				
	Proline dehydrogenase 1, mitochondrial* ** ***	149.81	1.479	0.004
	Glutamate dehydrogenase 1, mitochondrial* ** ***	376.49	1.395	0.006
Alpha-amino acid metabolic process*, cellular amino acid metabolic process*				
	CAD protein ** ***	59.22	1.389	0.008
	Thiosulfate sulfurtransferase ** ***	105.85	1.377	0.008
Carboxylic acid metabolic process**, oxoacid metabolic process**, organic acid metabolic process**				
	Beta-glucuronidase ***	7.602	2.511	2.43E-05
	Carnitine O-palmitoyltrans-ferase 1, liver isoform***	100.58	1.354	0.024
	Pyruvate carboxylase, mitochondrial	542.37	1.458	0.002
Small molecule metabolic process***				
	Succinate-CoA ligase [GDP-forming] subunit beta, mitochondrial	80.578	1.851	0.005
	Hydroxymethyl-glutaryl-CoA synthase, mitochondrial	34.06	1.817	0.0010
	Triokinase/FMN cyclase	8.467	1.496	0.0266
	Transthyretin	48.964	2.494	1.009E-06

Overexpressed proteins for glutamate metabolism in the white matter of hyh mice. *; **, *** Proteins in the classes are included in the respective child classes (see Table 8). For meaning of sum PEP score see Table 2 legend

In the case of overexpressed proteins, there was substantial correlation with ribosome-related and mitochondrial ribosome-related pathways (Fig. 6). This was corroborated by analysis of the PANTHER reactome pathway database, which is a relational database of signalling and metabolic molecules and their relations organized into biological pathways and processes [40]. This analysis revealed upregulation of proteins related to RNA and protein metabolism in the white matter of hyh mice (Table 10). These results suggest the presence of cell reactions that could include the glial responses that are involved in hydrocephalus pathology [11, 41].

**Table 10:** Reactome pathways

Reactome pathways	Number of genes process	Number of genes overexpressed	Expected value	Fold enrich-ment	P-value
Formation of the ternary complex, and subsequently, the 43S complex (R-MMU-72695)*	58	17	0.41	41.89	2.79E-19
Ribosomal scanning and start codon recognition (R-MMU-72702)*	59	22	0.41	53.22	7.84E-28
Translation initiation complex formation (R-MMU-72649)*	59	22	0.41	53.22	7.84E-28
Activation of the mRNA upon binding of the cap-binding complex and eIFs, and subsequent binding to 43S (R-MMU-72662)	60	22	0.42	52.33	1.13E-27
SRP-dependent cotranslational protein targeting to membrane (R-MMU-1799339)*	90	25	0.63	39.65	8.06E-29
Formation of a pool of free 40S subunits (R-MMU-72689)*	99	27	0.69	38.92	4.01E-31
Nonsense mediated decay (NMD) independent of the Exon Junction Complex (EJC) (R-MMU-975956)**	92	25	0.64	38.78	1.38E-28
L13a-mediated translational silencing of Ceruloplasmin expression (R-MMU-156827)*	109	27	0.76	35.35	5.09E-30
GTP hydrolysis and joining of the 60S ribosomal subunit (R-MMU-72706)*	110	27	0.77	35.03	6.48E-30
Cap-dependent translation initiation (R-MMU-72737)	117	27	0.82	32.94	3.30E-29
Nonsense mediated decay (NMD) enhanced by the Exon Junction complex (EJC) (R-MMU-975957)**	113	25	0.79	31.58	2.09E-26
Nonsense-mediated decay (NMD) (R-MMU-927802)**	113	25	0.79	31.58	2.09E-26
Mitochondrial translation elongation (R-MMU-5389840)*	86	19	0.60	31.53	1.69E-19
Mitochondrial translation termination (R-MMU-5419276)*	88	19	0.62	30.82	2.59E-19
Mitochondrial translation (R-MMU-5368287)*	89	19	0.62	30.47	3.19E-19
Translation (R-MMU-72766)	225	46	1.58	29.18	7.49E-50
Nonsense mediated decay (NMD) enhanced by the Exon Junction Complex (EJC) (R-MMU-975957)**	113	25	0.79	31.58	2.09E-26
Major pathway of rRNA processing in the nucleolus and cytosol (R-MMU-6791226)**	175	25	1.23	20.39	8.25E-22
rRNA processing in the nucleus and cytosol (R-MMU-8868773)**	175	25	1.23	20.39	8.25E-22
rRNA processing (R-MMU-72312)**	175	25	1.23	20.39	8.25E-22
Metabolism of RNA (R-MMU-8953854)**	566	26	3.97	6.56	6.37E-11
Metabolism of proteins (R-MMU-392499)*	1644	59	11.52	5.12	1.03E-23
Unclassified (UNCLASSIFIED)	12,982	38	90.96	0.42	0.00E00

Reactome pathways associated with the overexpressed proteins in the white matter of hydrocephalic hyh mice identified by the PANTHER data classification system. Binomial analysis with Bonferroni correction for multiple testing with corrected p < 0.05. * metabolism of proteins; ** metabolism of RNA

**Fig. 7:**
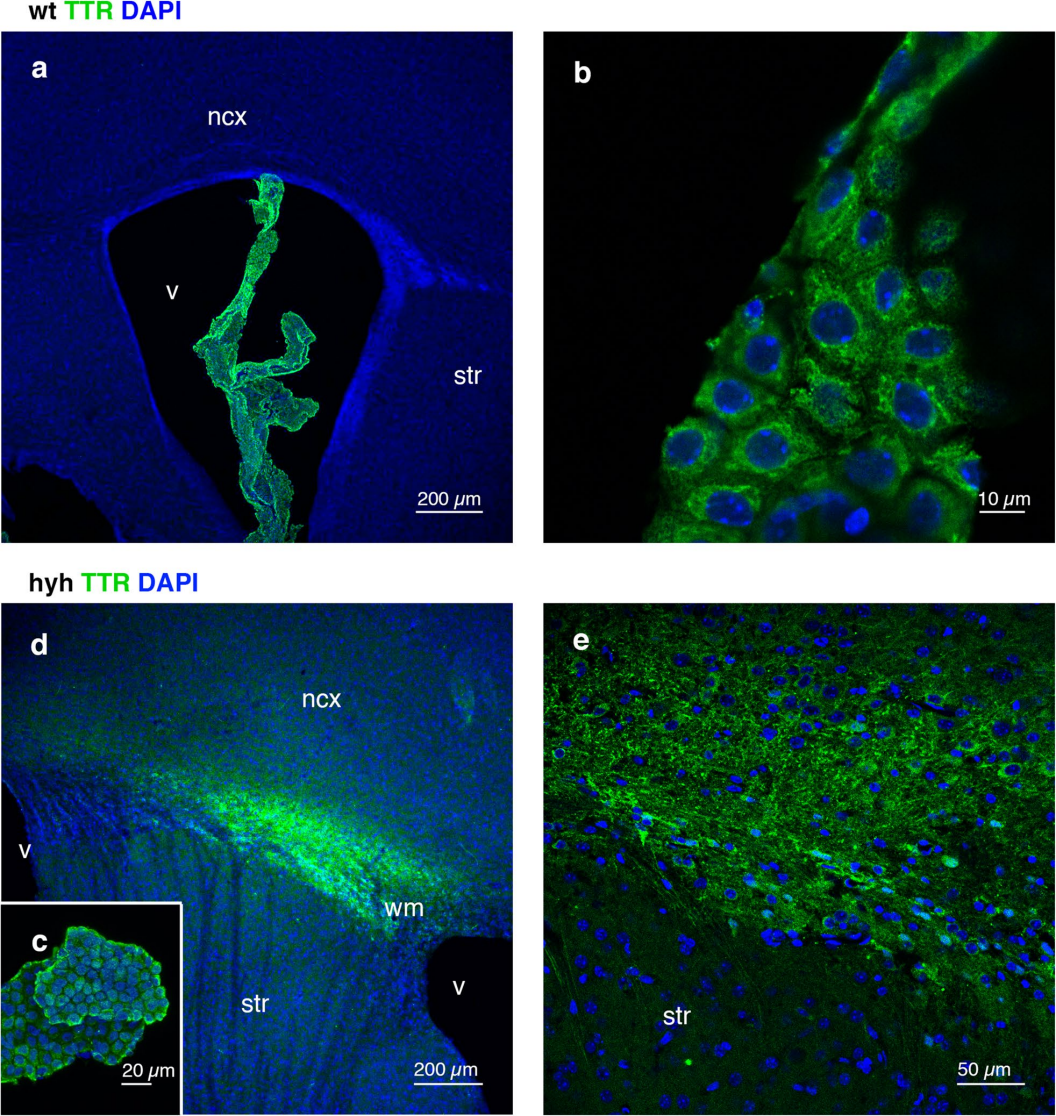
Expression of transthyretin (TTR) in the choroid plexus and white matter. Immunofluorescence for TTR in frontal sections of normal (wt, **a, b**) and hydrocephalic hyh (**c–e**) mice. Choroid plexus epithelial cells expressed TTR in wt (**a** and **b**) and hyh (**c**) mice. Unlike those in normal mice, cells in the affected white matter of hyh mice also expressed TTR (**d** and **e**). *ncx* neocortex, *v* lateral ventricle lumen, *str*, striatum, *wm* white matter. The nuclei are stained *blue* with DAPI

Transthyretin (TTR) was also overexpressed in the white matter of hyh mice (Fig. 6). TTR is a protein with neuroprotective effects that is secreted into the cerebrospinal fluid (CSF) by the choroid plexus and the subcommissural organ [42]. Interestingly, OPCs use TTR for their differentiation [43]. For this reason, TTR was immunodetected in tissue sections, and the protein was found to be present in the choroid plexus of normal and hydrocephalic mice (Fig. 7a–c). Interestingly, TTR was also detected in the affected white matter in hyh mice but not in normal mice (Fig. 7a, d). TTR immunolabelling was not detected in the brain parenchyma of different ventricle walls where ependyma was not present [17, 23] or in the meningeal surface, as shown in the Additional file 4. Only a few cells in the ventricle surfaces showed punctate labelling for TTR, which can be suggested as reactive astrocyte endocytosis from the CSF, according to the study by Roales-Buján et al*.* [23].

Finally, an additional ad hoc analysis of the proteome (Table 11) including proteins selected according to references further confirmed the defects in oligodendrocyte development and differentiation and the presence of reactive astroglia in the white matter.

**Table 11:** Functionally significant underexpressed and overexpressed proteins

Gene name	Sum PEP Score	Abundance Ratio: (Sample) / (Control)	Abundance Ratio P-Value: (Sample) / (Control)
Aldehyde dehydrogenase family 1 member A3	9.87	3.63 ( >)	2.42E-07
Breast carcinoma-amplified sequence 1 homolog	79.33	0.51 ( <)	5.41E-06
Dynactin subunit 1	430.29	1.849 ( >)	1.44E-06
Dynactin subunit 4	108.84	1.58 ( >)	0.0003
Ermin	56.84	0.51 ( <)	0.0001
Galectin 1	21.94	1.61 ( >)	0.012
Gap junction gamma-3 protein	18.27	0.32 ( <)	9.09E-08
Gap junction alpha-1 protein	59.62	2.52 ( >)	1.20E-07
Gelsolin	178.38	0.72 ( <)	0.03
Neural cell adhesion molecule 1	542.26	1.62 ( >)	0.0001
Pleiotrophin	19.36	1.47 ( >)	0.03

Functionally significant underexpressed ( < ) and overexpressed ( > ) proteins in the white matter of hyh mice related to oligodendrocyte differentiation and glial reactions that may be involved in hydrocephalus pathology. For meaning of sum PEP score and PSM see Table 2 legend

### Analysis of oligodendrocyte progenitor cells in the white matter

**Fig. 8:**
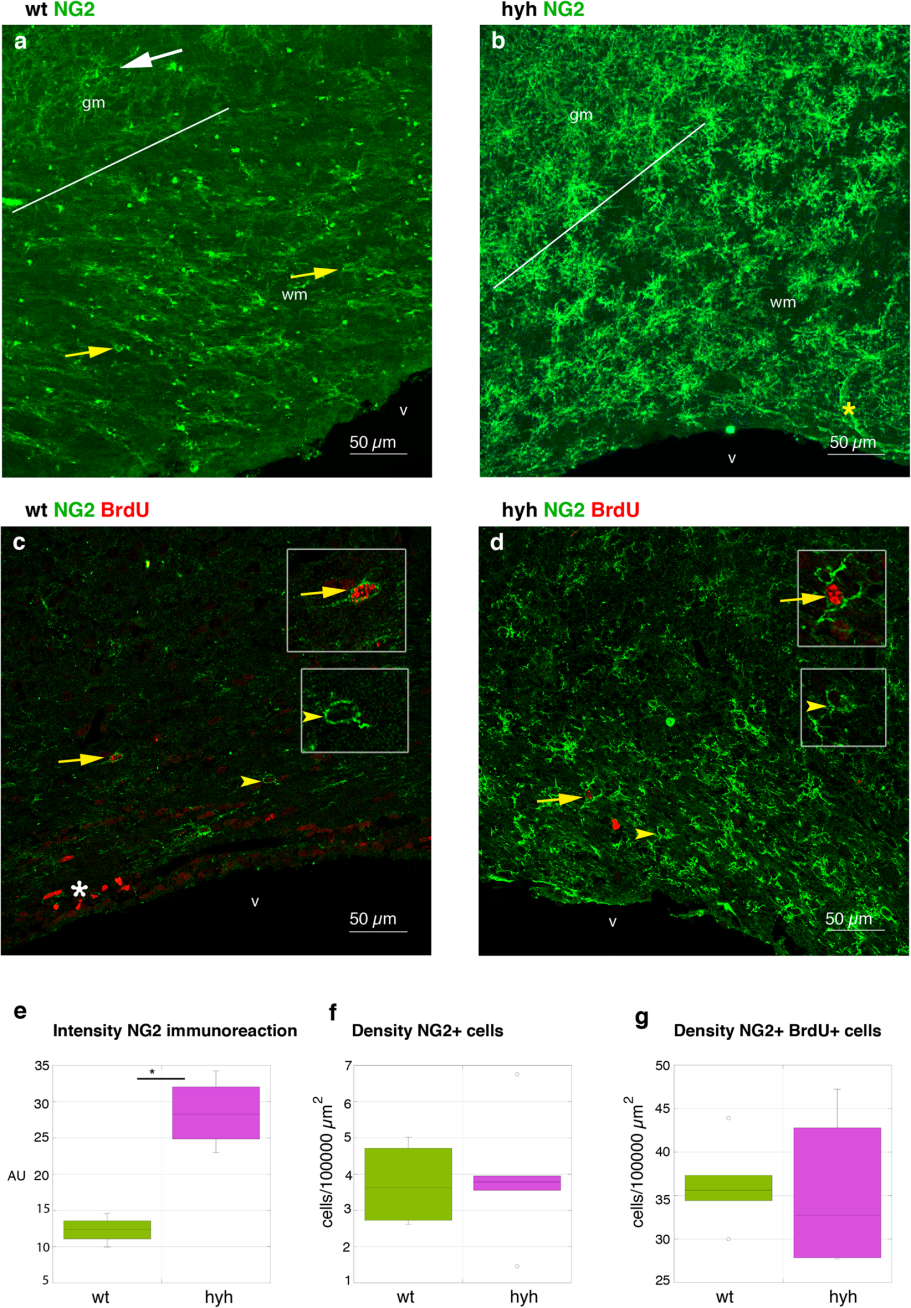
Reactive oligodendrocyte progenitor cells (OPCs) in the white matter of hydrocephalic hyh mice. **a–d** Expression of the NG2 antigen in OPCs in neocortical frontal sections of normal (*wt*) and hyh mice. Immunofluorescence for NG2 (*green*) and bromodeoxyuridine (*BrdU*, *red*) in frontal sections of wt (**a**, 42 µm Z projection; **c**, 1 µm plane) and hyh (**b**, 31 µm Z projection; **d**, 1 µm plane) mice. The *white* and *yellow arrows* in **a** indicate NG2 cells in the grey and white matter, respectively. NG2 cells presenting BrdU labelled nuclei (*arrows* in **c** and **d**) and lacking BrdU labelling (arrowheads in **c** and **d**) can be observed (details in *insets*). Pericytes (*yellow asterisk* in **b**) also exhibited NG2 labelling. The lines in **a** and **b** indicate the border between white and grey matter. In wt mice proliferating neuroblasts in the subventricular zone presented BrdU labelling (*white asterisk* in **c**). **e** Intensity of NG2 immunoreactivity (*AU*, arbitrary units) in the white matter of wt ( n = 4) and hyh (n = 4) mice. **f, g** Density of OPCs labelled with NG2 in the white matter of wt and hyh mice without (**f**) (wt, n = 4; hyh n = 4) and after (**g**) (wt, n = 4; hyh n = 5) a pulse of BrdU. *p < 0.05; the Wilcoxon-Mann–Whitney test. *gm* grey matter, *v* lateral ventricle lumen, *wm* white matter

**Fig. 9:**
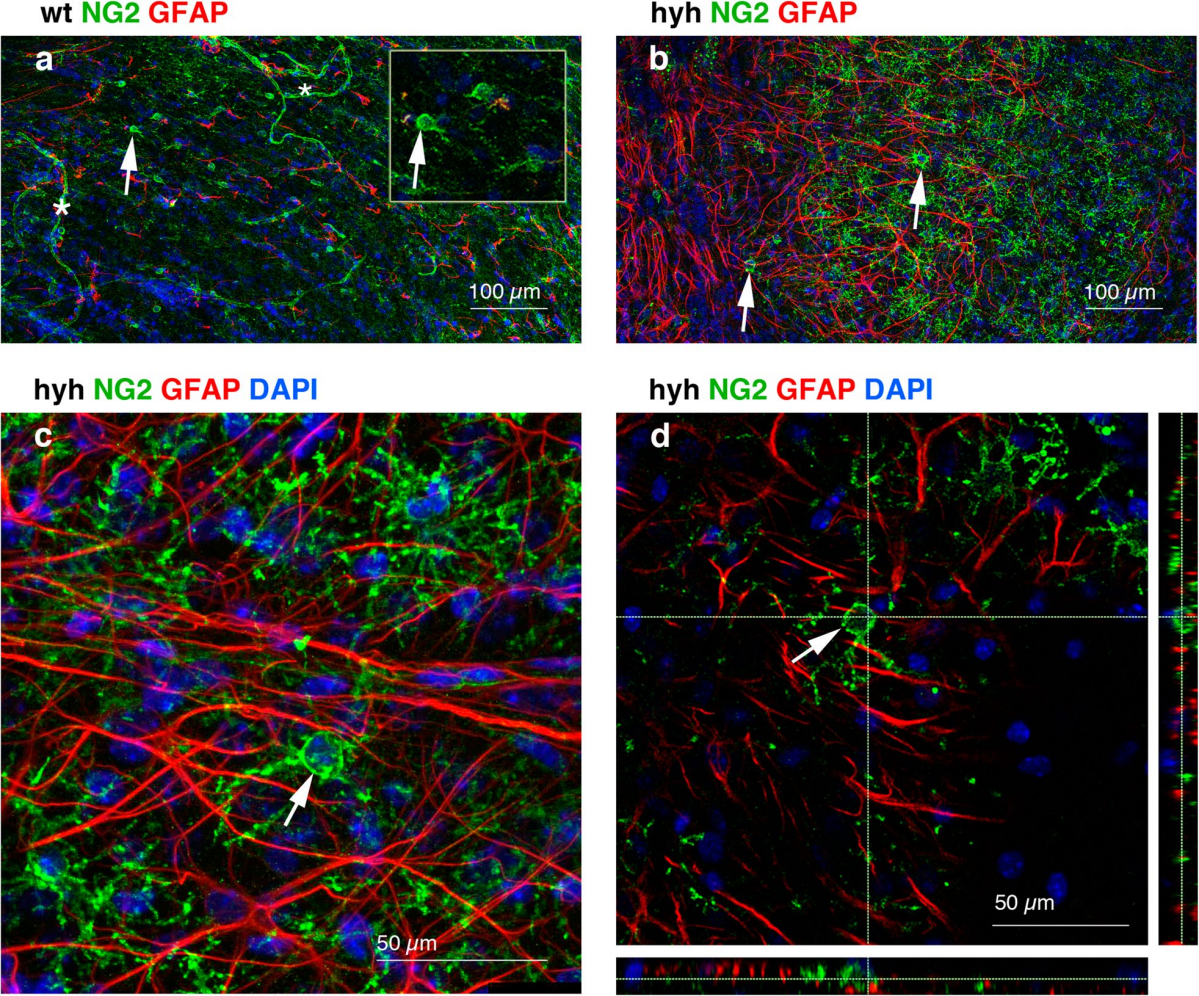
Reactive NG2-expressing cells are not astrocytes. **a–e** Immunofluorescence staining for NG2 (*green*) and GFAP (*red*) in whole-mounts of the neocortex from normal (*wt*, **a**) and hyh (**b-d**) mice. **a** Sixty-seven micron projection. NG2 labelling of reactive oligodendrocyte progenitor cells (OPCs, *arrows*, detailed in *inset*) and pericytes in capillaries (*asterisk*). Pericytes (*asterisks*) exhibited NG2 labelling. **b, c** Thirty-four micron Z projections. **c** In the hyh mouse, OPCs were labelled with NG2 but not with GFAP (*arrows* in **b** and **c**). **d** Pseudo 3D reconstruction (28-µm-Z projection) showing an OPC (*arrow*) labelled with NG2 and immunonegative for GFAP. The nuclei were stained *blue* with DAPI

Remarkably, analysis of protein expression in the white matter revealed defects in the processes of oligodendrocyte cell development and differentiation, whereas the NG2 antigen, which is present in OPCs, was overexpressed. To uncover the cause of this phenomenon, OPCs were further studied because several in vivo and in vitro studies have demonstrated the ability of NG2 cells to differentiate into oligodendrocytes [44–49]. In the neocortical white matter of hyh mice, compared with wt mice, immunolabelling with anti-NG2 showed a stronger immunoreaction in the white matter in hyh mice than in wt mice, but not a higher density of OPCs (Fig. 8a, b, e, f). While in wt mice, NG2 was restricted to the cell bodies of OPCs, in hyh mice, immunoreactivity was detected in the profusely ramified cell processes of OPCs. In addition, in wt mice, OPCs labelled with NG2 exhibited a fusiform morphology and were oriented according to the direction of the myelin fibres in the white matter (Figs. 8a and 9a). NG2 labelling could also be detected in the pericytes of normal and hyh mice (Figs. 8b and 9a). The density of NG2 cells labelled with BrdU was not significantly different between hyh and wt mice (Fig. 8c, d, g), thus excluding a higher rate of NG2 cell proliferation for a 24-h period.

**Fig. 10:**
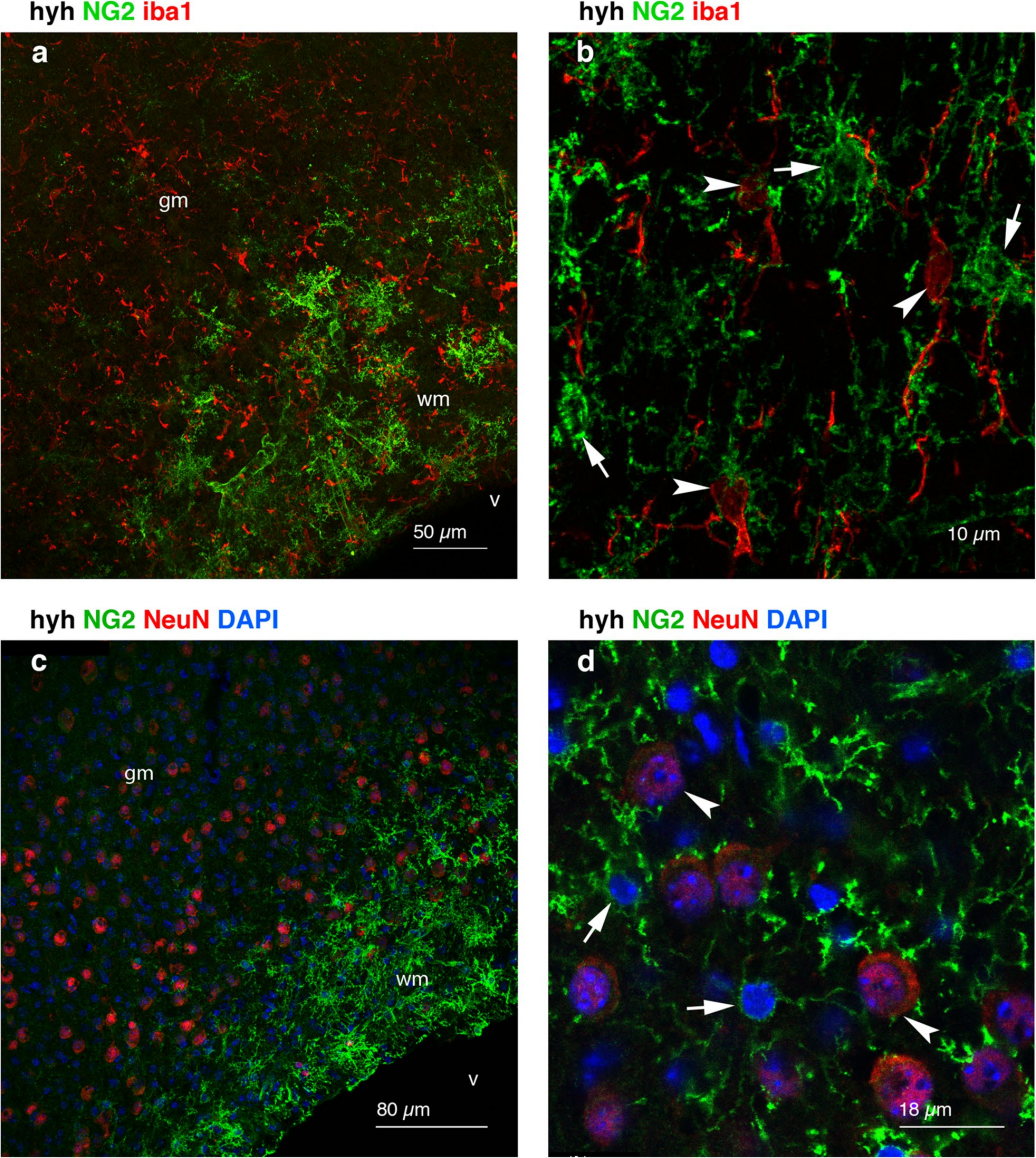
NG2-expressing cells are not microglia or neurons. **a, b** Frontal sections of the neocortex from a hydrocephalic hyh mouse immunostained for NG2 (*green*) and Iba1 (*red*). Seventy-two micron Z projection. Microglial cells were immunonegative for NG2 (*arrowheads*). NG2 cells are indicated with *arrows*. **c, d** Frontal sections of the neocortex from a hyh mouse immunostained for NG2 (*green*) and NeuN (*red*) (18 µm Z projection in **c**). Details in a 1 µm-thick plane are shown in **d**. NG2 cells (*arrows* in **d**) present smaller nuclei compared to neurons (*arrowheads* in **d**)*.* *gm* grey matter, *v* lateral ventricle lumen, *wm* white matter. The nuclei were stained *blue* with DAPI

Although it has been speculated that NG2 cells can generate astrocytes under conditions of central nervous system injury, most studies have discarded this hypothesis [50]. Accordingly, our results showed that NG2-positive cells located in the white matter of the hyh hydrocephalic mice were not GFAP-positive, thus indicating that these cells were not reactive astrocytes (Fig. 9). Following iba1 (Fig. 10a, b) and NeuN (Fig. 10c, d) staining, cells labelled with NG2 were immunonegative for these microglia and neuron markers, respectively, and thus NG2 cells were also discarded as those cell types. Thus, according to these results, in the present study, NG2-positive cells can be considered unequivocally OPCs, in agreement with previous works [46].

**Fig. 11:**
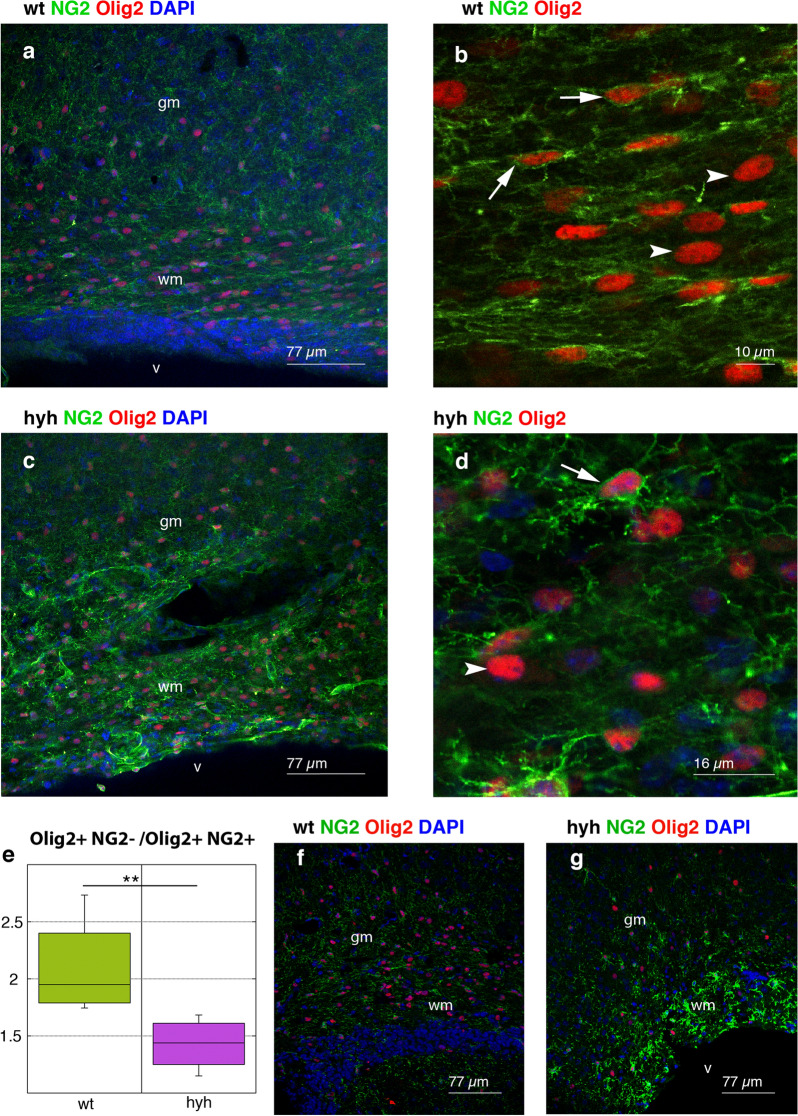
Density of mature oligodendrocytes in the white matter. Frontal sections of the neocortex of normal (*wt*; **a, b**) and hydrocephalic hyh (**c, d**) mice immunostained for NG2 (*green*) and Olig2 (*red*). OPCs that were positive for NG2 also exhibited Olig2 staining in their nuclei (*arrows* in **b** and **d**). Mature oligodendrocytes were labelled with Olig2 but not NG2 (*arrowheads* in **b** and **d**). **a** and **c** correspond to 17 and 28 µm Z protections, respectively. **e** The proportion of mature oligodendrocytes (Olig2-positive and NG2-negative) relative to OPCs (Olig2-positive and NG2-positive) in the white matter of wt (n = 6) and hyh (n = 6) mice. **f**, **g** Representative images (1 µm thick planes) used for quantification in **e**. **p < 0.005; the Wilcoxon-Mann–Whitney test. *gm* grey matter, *v* lateral ventricle lumen, *wm* white matter. The nuclei were stained *blue* with DAPI

In the white matter of hyh mice, the proportion of mature oligodendrocytes versus OPCs was calculated to detect whether reactive NG2-positive OPCs differentiated into mature oligodendrocytes. Olig2 is a reliable marker of both mature oligodendrocytes [51] and OPCs what was corroborated in the present study in both wt and hyh mice (Fig. 11a–d). In contrast, NG2 was expressed in OPCs but not in mature oligodendrocytes (Fig. 11a–d). Thus, cells labelled with NG2 and Olig2 were OPCs, and mature oligodendrocytes were NG2-negative and Olig2-positive. Cell quantification revealed a lower proportion of mature oligodendrocytes than OPCs in the white matter in hyh mice than in wt mice (Fig. 11e-g, see also the Additional file 5). Therefore, in hyh mice, reactive OPCs overexpressed NG2 but did not appear to give rise to a higher density of mature oligodendrocytes.

## Discussion

Direct or indirect damage to periventricular neuronal axons is a common neuropathological event in congenital hydrocephalus [52]. However, neurons of the neocortical grey matter affected by this neuropathological condition cannot be easily detected through common observation using light microscopy [41], which could be why the effects on neurons are considered negligible compared to those on oligodendrocytes [41, 52]. Neuronal damage associated with oedema has been experimentally supported by bioenzymatic data [4]. In the present study, microscopic observation of the cerebral cortex of hyh mice has indicated that the injured white matter exhibits an oedematous appearance, as in other forms of congenital hydrocephalus, including in humans [41, 53]. However, in the grey matter, it is not easy to detect such effects. In the cerebral white matter of hyh mice, we found evidence of oedema, as indicated by an increase in sodium and chloride levels and a decrease in potassium levels, which extended to the grey matter. Thus, assessment of oedema conditions by EDS-SEM can be a useful method to test the effects of therapies on the brain parenchyma, especially near barriers. According to these results, it was considered necessary to identify biomarkers of damage in the grey matter, where analysis of the lipid profile by MALDI-MSI has provided promising results.

### The grey matter presents a lipid profile that may be an indicator of neural dysfunction

Lipid metabolism may be of particular importance for the central nervous system, as it involves the second highest concentration of lipids, exceeded only by adipose tissue. The crucial role of lipids in cell signalling and tissue physiology has been demonstrated in many neurological disorders [54]. Biosynthetic pathways for phospholipid production appear to operate through different cellular mechanisms; thus, the composition of phospholipids may reflect different cellular states.

In the neocortical grey matter of hyh mice, we found marked underexpression of two PI molecular species, with 34:4 and 34:5 fatty acyl chains, which may indicate alterations in neural function. The fatty acyl chains 34:4 and 34:5 have been reported to be dominant components in the rodent and human brains [55]. This finding is the consequence of the activity of enzymes involved in the synthesis, remodelling, and substrate preferences of these fatty acids [55]. Inositol lipids are enriched in the brain, where they support key cellular functions, and alterations in these molecules could lead to diseases of the nervous system [56]. PI is an essential phospholipid with a role in membrane structures because it is used to synthesize phosphoinositides, inositol polyphosphates, and complex sphingolipids and is catabolized to glycerophosphoinositol [57]. PI and its metabolites regulate a diverse set of cellular processes, such as glycolipid anchoring of proteins, signal transduction, mRNA export from the nucleus, vesicle trafficking, and cytoskeleton dynamics, and serve as reservoirs of second messengers [57, 58]. Serum levels of PI (38:4) and PI (38:5) in relation to other lipids are considered biomarkers of post-ischaemic cognitive impairment in rats [59]. The lower levels of PI in hyh mice in the present study are consistent with the levels of inositol found in a study of this animal model by ^1^H high-resolution magic angle spinning nuclear magnetic resonance (^1^H HR-MAS NMR) spectroscopy [19]. The pathological meaning of the lower levels of PI in the hydrocephalic hyh mouse is not clear. In our results, PI appear to be mainly present in the neurons (see Fig. 4e, f, i, j). On the other hand, astrocytes are considered a source of inositol [60]. Still, the presence of an astrocyte reaction in the hydrocephalic hyh mouse is not in agreement with the observed lower levels of inositol. Therefore, these lower levels of inositol can be suggested to be more related to neuronal dysfunction in the neocortical grey matter.

It should be pointed out that differences in the lipid species concentration were not detected in the white matter when comparing wt and hydrocephalic hyh mice. Thus, in the hyh mice, the effect on white myelin was not reflected in the composition of PS and PI molecular species. Testing the changes in lipids in the white matter will probably need another experimental approximation as MALDI-MSI with positive ion mode detection.

### Possible biomarkers of cell effects in the cerebral grey and white matter

In the neocortical grey matter of hyh mice, diacylglycerophosphoserine PS (42:9), which contains 20:3 and 22:6 fatty acyl chains, was overexpressed. PS is flopped from the inner plasma membrane leaflet to the outer leaflet by scramblase to give an eat-me signal in apoptotic cells. However, probably this would not explain why there are higher PS levels in the tissue. Also, it is not probable that such notable changes in phosphatidylserine levels detected in the present study were implied in apoptosis. We have previously shown that the apoptosis rate in the neocortical grey matter of the hyh mice is low, non-detectable [61]. Thus, apoptosis only can be detected in the periventricular white matter of hyh mice. On the other hand, evidence has been reported that PS (42:9) is implicated in a neuroprotective response. In the plasma membrane, PS forms part of the protein docking sites needed for activation of pro-survival and pro-growth protein kinase C, Raf1, and Akt signalling [62]. In the grey matter of the human brain, docosahexaenoic acid (DHA) (22:6) is the most abundant fatty acid [62]. DHA facilitates PS signalling [62–64] and promotes neuronal survival, neurogenesis, neurite development, neuronal cell migration, and synaptogenesis [65]. In addition, PS administration has been shown to have a neuroprotective effect in a mouse model of familial dysautonomia [66].

The overexpression of TTR in the white matter of hyh mice deserves attention because it could have a neuroprotective effect. Besides, TTR also promotes oligodendrocyte myelination from OPCs [43, 67]. TTR is a distributor of thyroid hormones [67] that is mainly synthetized in the choroid plexus and released into CSF, where it is one of the more abundant proteins [68]. TTR is also secreted by the subcommissural organ [42, 69]. The present results suggest that TTR can be produced in the damaged brain parenchyma or taken up by the white matter cells from the ventricle CSF. According to the absence of TTR found in different ventricle walls (see Additional file 4), a diffusion of TTR from the surfaces of the ventricles without ependyma appears to be improbable. However, the possibility of TTR diffusion in the oedematous white matter should not be discarded, where the OPCs could take the protein [43]. In any case, there is the possibility that TTR in the damaged white matter can be related to OPC function and oligodendrocyte development [43]. TTR has been implicated in stroke and ischaemia, in which it is overproduced by the choroid plexus to control neuronal cell death, oedema, and inflammation [70–73]. Therefore, it can be suggested that the source of TTR in the white matter could be the choroid plexus, the subcommissural organ, or even affected periventricular neurons and OPCs, but this topic requires further investigation. In any case, TTR can be suggested as a biomarker of parenchymal changes in hydrocephalus.

### The proteome profile of hydrocephalic mice can be an indicator of the role of reactive astroglia

In the hyh mouse, the astrocyte reaction has been profusely described. It has been associated with barrier properties and playing a role in the neural metabolism [17, 19, 20, 23]. The results of proteome analysis of the white matter of the hyh mouse are consistent with the presence of reactive astrocytes and possibly with their pivotal function. Accordingly, the gap junction alpha-1 protein (connexin Cx43), which is a protein mainly present in astrocytes, was overexpressed. In addition, several mitochondrial ribosomal proteins and other enzymes related to glutamate metabolism and astrocyte metabolism were overexpressed. Astrocytes are of critical importance for the biosynthesis of glutamine and GABA through the enzymes glutamine synthetase and pyruvate carboxylase [74]. In the present study, both enzymes were overexpressed in hyh mice. Thus, the present results agree with the high levels of glutamine previously observed in the neocortical grey and white matter of hyh mice by 1H HR-MAS NMR spectroscopy [19]. Astrocytes also use pyruvate carboxylase for anaplerotic metabolism to replenish tricarboxylic acid cycle intermediates [75]. Under physiological and pathophysiological conditions, mitochondria are considered to play relevant roles in the central nervous system. In the case of glial cells, mitochondria are related to their metabolism and cooperation with neurons [76]. Therefore, the present results provide evidence of a possible influence of reactive astrocyte metabolism in the affected brain parenchyma.

### Oligodendrocyte differentiation is affected in the white matter

Proteome analysis revealed demyelination and possible defects in oligodendrocyte differentiation in the white matter of hyh mice, suggesting that OPCs may have been affected. The possibility of contamination of the white matter with grey matter could not be excluded. However, the study of the results in tissue sections with different cell markers and immunofluorescence, such as the NG2 cells and TTR results, strengthen the quality of the isolation procedure.

Among the underexpressed proteins that indicate demyelination in the hyh mice are gap junction gamma-3 protein (connexin Cx29), which is present in oligodendrocytes [77], breast carcinoma amplified sequence 1 homologue (BCAS1), and ermin. The observed lower Cx29 expression was in contrast with the higher levels of the gap junction alpha-1 protein (Cx43) characteristic of astrocytes [77] (see above). BCAS1 has been described to be specifically expressed in oligodendrocytes and Schwann cells, and a decrease in its expression indicates demyelination [78]. Ermin is a myelinating oligodendrocyte-specific protein that regulates cell morphology. Ermin is considered a marker of myelinating oligodendroglia and probably plays a role in cytoskeletal rearrangement during the late wrapping and/or compaction phases of myelinogenesis [79].

However, in contrast, the present investigation revealed changes in the regulation of some proteins that could indicate stimulation of the oligodendrocyte differentiation process. This is the case for dynactin and NCAM, which were overexpressed in the white matter of hyh mice. Dynactin is necessary for anterograde transport of myelin basic protein mRNA in oligodendrocytes and myelination in vivo [80]. NCAM promotes oligodendrocyte differentiation and myelin repair [81]. In addition, the decreased levels of gelsolin that we detected in the white matter of hyh mice indicate OPC stimulation. Gelsolin works downstream of leucine-rich repeat and Ig-like domain-containing Nogo receptor-interacting protein 1 (LINGO-1) [82]. LINGO-1 is a negative regulator of OPCs differentiation [82].

Additionally, the present investigation also revealed changes in the expression of other proteins in hyh mice that indicate alterations in the differentiation of oligodendrocytes. Retinoic acid is a morphogen synthesized from retinaldehyde through oxidation by retinaldehyde dehydrogenase, an enzyme that, in the present study, was overexpressed in the white matter of hyh mice. Retinoic acid induces oligodendrogenesis in the striatum subventricular zone [83] but inhibits the maturation of embryonic spinal cord oligodendrocyte precursors [84]. The levels of galectin-1 were higher in the white matter of hyh mice than that of wt mice. Galectins are very potent regulators of neuroinflammation, and their secretion also results in modification of developmental myelination and remyelination [85].

In the present study, a key result is that OPCs are overexpressing the NG2 antigen. It is expected that the role of reactive OPCs in hydrocephalus is remyelination through oligodendrocyte differentiation, but as it is discussed below, conditions could not be appropriate. It is relevant to highlight that the density of NG2 cells is similar between wt and hyh mice. Therefore, the consequence of αSNAP mutation appears not to affect the development of NG2 cells. If there is any chance of alteration in the progenitor fate during perinatal oligodendrogenesis due to the αSNAP mutation, a change in the numbers of oligodendrocytes would be expected. However, only a difference in the expression of NG2 is detected but not in the density of cells. There is also the possibility of a delayed maturation of OPCs, which can be inferred from the observed lower density of mature oligodendrocytes in the hydrocephalic mice. Proteome analysis of hyh mice showed overexpression of proteins implicated in the phosphatidylcholine metabolism, one of the main components of brain cell membranes [86]. Besides, reactome analysis indicated stimulation of protein biosynthesis. All these processes may be related to the response of OPCs but also the reactivity or changes in other cells, such as astrocytes, endothelial cells, and axons. A decrease in myelin levels in hydrocephalus could trigger the proliferation and differentiation of OPCs to counter the loss of myelin in the CNS [52]. However, our results indicate failure of this process. OPCs are capable of generating myelinating oligodendrocytes after acute demyelination, but probably not under chronic demyelinating conditions [44, 87]. In chronic injury, OPCs function appears to be limited by a hostile environment or by the inability of OPCs to proliferate further and differentiate [87].

If the function of reactive OPCs is to produce myelinating oligodendrocytes, there are possible explanations for their failure. First, in the fully developed brain, compared with the perinatal stages, OPCs have a lower rate in producing myelinating oligodendrocytes [88]. It has been suggested that the availability of axons to be myelinated might be a cause of this phenomenon [88]. However, although the PANTHER analysis did not give axon damage information, the damage should not be discarded according to evidence presented in other animal models [2–6]. Second, the environment in the white matter could be unfavourable for the production of myelinating oligodendrocytes. This explanation fit better with the present and previous results. The neocortex of hyh mice express high levels of neurocytotoxic glutamate [19] and TNFα [20]. The results of the present study related to the expression of proteins related to glutamate metabolism are consistent with these high levels. It is known that glutamate [89] and reactive oxygen species [90, 91] induce excitotoxic cell death associated with white matter damage.

### Role of NG2 in the injured white matter

In the present study, reactive OPCs overexpressed the NG2 antigen. In this case, the role of reactive OPCs can be explained based on the functions of the NG2 proteoglycan. The processing of the NG2 molecule is implicated in several biological functions, such as neuromodulation, protection of OPCs from apoptosis, OPC migration, gene expression, and adhesion [29]. It has been reported that NG2 expression appears to be regulated by inflammation through TNFα and hypoxia-induced signal transduction [92]. In addition, TNFα triggers oligodendrocyte apoptosis, thus affecting myelination, and inhibits OPC proliferation and differentiation [93]. In human foetuses with hydrocephalus and in hyh mice, there are high levels of the proinflammatory cytokine TNFα, which is associated with reactive astrocytes in the periventricular white matter [20]. Thus, some of these conditions could trigger the observed NG2 overexpression and affect OPCs behaviour.

### Conclusions

In the cerebral cortex of hyh mice with obstructive hereditary congenital hydrocephalus, these results support the presence of astrocyte reaction and alterations in the white matter, both common events previously described in experimental animal models of hydrocephalus and human cases [1, 41, 94, 95]. Additionally, EDS-SEM showed that hydrocephalic oedema extends further from the white matter towards the grey matter. NG2 antigen, TTR, and PI and PS molecular species of lipids have been found as possible biomarkers for the pathogenesis of hydrocephalus. These molecules may also be useful to find biomarkers to test the beneficial effect of experimental therapies. In the grey matter, PI and PS molecular species are lipids that can be related to neuronal damage. In the white matter, which exhibits defective oligodendrocyte development and differentiation processes, there was evidence of an OPC reaction. These reactive OPCs overexpress NG2 antigen and they could be triggered by inflammatory and neurocytotoxic conditions, hypoxia, and neurodegeneration. It is unknown if the *Napa* mutation in the hyh mouse, which affects neuroprogenitor cell development, influence these biomarkers. Therefore, further research of these molecules and the OPCs, including the study in other animal models, will be helpful to understand their role in the pathogenesis.

## Supplementary Information

**Additional file 1:** View of the white matter (*arrow*) exposed in the ventricle of a hyh mouse to be dissected out.

**Additional file 2: **Atomic percentage of calcium detected by EDS-SEM. Description of data: Atomic percentage of calcium is represented in the white matter (*WM*) and grey matter (*GM*) of normal (*wt*) (n = 3) and hydrocephalic hyh (n = 3) mice. No significant differences are present (Student’s t-test).

**Additional file 3:** Overexpressed proteins as indicators of astrocyte reaction detected by UHPLC–HRMS. Abundance ratios (Sample hyh) / (Control wt) for GFAP (Gfap, glial fibrillary acidic protein) and vimentin (vim) (wt, n = 4; hyh, n = 5). The sum PEP score corresponds to the score calculated based on the posterior error probability (PEP) values of the peptide spectrum matches (PSM). The PEP indicates the probability that an observed PSM is a random event. Sum PEP score is calculated as the negative logarithms of the PEP values of the connected PSM.

**Additional file 4:** Immunolabelling of TTR in different ventricle walls of hyh mice. Description of data: TTR was not found diffusing towards the different walls lacking the ependyma barrier, such as in the lateral ventricle in its dorsal (**a**; **b** is a detail of **a**) and lateral walls (**c**), nor in the third ventricle (**e**; **f** is a detail of **e**). The cerebral surface containing meninges also lack of TTR labelling (**d**). In the ventricle surfaces, labelling pattern of some cells (arrows in **a**, **b**, **e**, and **f**) covering the ventricle surface suggests endocytocis by periventricular astrocytes, as reported by Roales-Buján et al. [23]. *ncx* neocortex, *str* striatum, *v* ventricle lumen.

**Additional file 5:** Percentages respect to total number of cells (labelled with DAPI) of mature oligodendrocytes (Olig2-positive and NG2-negative) relative to OPCs (Olig2-positive and NG2-positive). This has been represented for the white matter of wt (n = 6) and hyh (n = 6) mice. * *p* < 0.05, Student’s t-test.

## Data Availability

The datasets used and/or analysed during the current study are available from the corresponding author on reasonable request.
