# Supplementary figures and images for: Identification of key molecular biomarkers involved in reactive and neurodegenerative processes present in inherited congenital hydrocephalus
Source: Fluids Barriers CNS. 2021 Jul 2;18:30. doi: 10.1186/s12987-021-00263-2 (PMC8254311; doi:10.1186/s12987-021-00263-2)

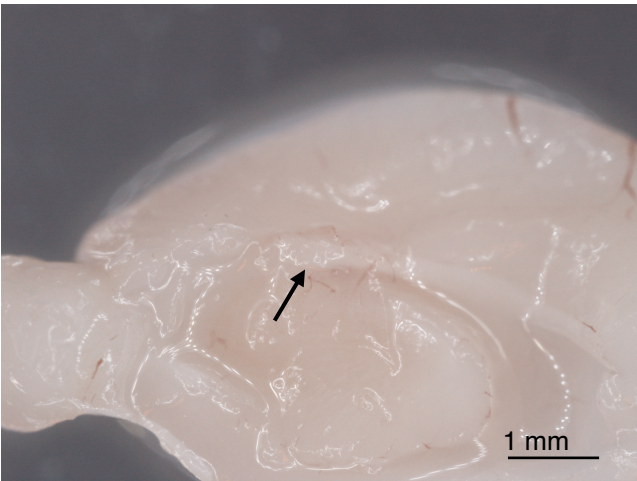

Additional file 1

Supplement: Supplementary file 1 — Additional file 1: View of the white matter (arrow) exposed in the ventricle of a hyh mouse to be dissected out. [file 12987_2021_263_MOESM1_ESM.pdf]

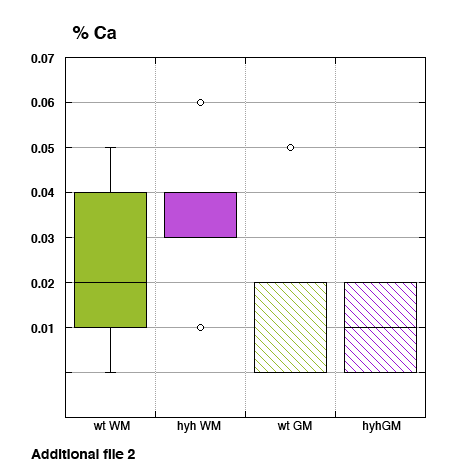

Supplement: Supplementary file 2 — Additional file 2: Atomic percentage of calcium detected by EDS-SEM. Description of data: Atomic percentage of calcium is represented in the white matter (WM) and grey matter (GM) of normal (wt) (n = 3) and hydrocephalic hyh (n = 3) mice. No significant differences are present (Student’s t-test). [file 12987_2021_263_MOESM2_ESM.tif]

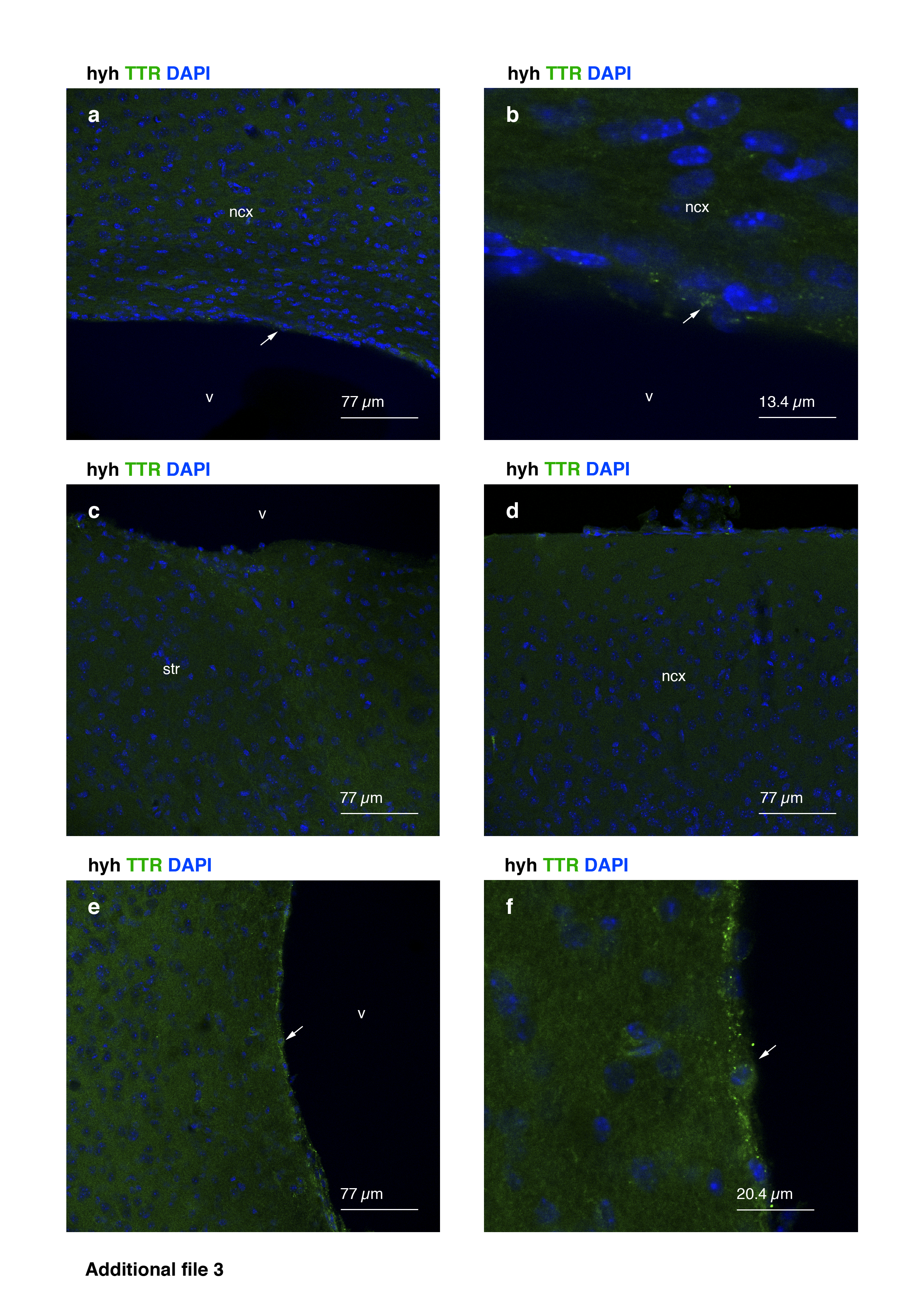

Supplement: Supplementary file 4 — Additional file 4: Immunolabelling of TTR in different ventricle walls of hyh mice. Description of data: TTR was not found diffusing towards the different walls lacking the ependyma barrier, such as in the lateral ventricle in its dorsal (a; b is a detail of a) and lateral walls (c), nor in the third ventricle (e; f is a detail of e). The cerebral surface containing meninges also lack of TTR labelling (d). In the ventricle surfaces, labelling pattern of some cells (arrows in a, b, e, and f) covering the ventricle surface suggests endocytocis by periventricular astrocytes, as reported by Roales-Buján et al. [23]. ncx neocortex, str striatum, v ventricle lumen. [file 12987_2021_263_MOESM4_ESM.tif]

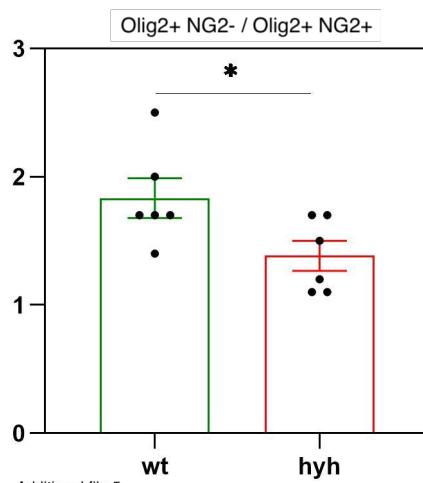

Additional file 5

Supplement: Supplementary file 5 — Additional file 5: Percentages respect to total number of cells (labelled with DAPI) of mature oligodendrocytes (Olig2-positive and NG2-negative) relative to OPCs (Olig2-positive and NG2-positive). This has been represented for the white matter of wt (n = 6) and hyh (n = 6) mice. * p < 0.05, Student’s t-test. [file 12987_2021_263_MOESM5_ESM.pdf]
