# Supplementary material for: Identification of key molecular biomarkers involved in reactive and neurodegenerative processes present in inherited congenital hydrocephalus
Source: Fluids Barriers CNS. 2021 Jul 2;18:30. doi: 10.1186/s12987-021-00263-2 (PMC8254311; doi:10.1186/s12987-021-00263-2)
Supplement: Supplementary file 3 — Additional file 3: Overexpressed proteins as indicators of astrocyte reaction detected by UHPLC–HRMS. Abundance ratios (Sample hyh) / (Control wt) for GFAP (Gfap, glial fibrillary acidic protein) and vimentin (vim) (wt, n = 4; hyh, n = 5). The sum PEP score corresponds to the score calculated based on the posterior error probability (PEP) values of the peptide spectrum matches (PSM). The PEP indicates the probability that an observed PSM is a random event. Sum PEP score is calculated as the negative logarithms of the PEP values of the connected PSM. [file 12987_2021_263_MOESM3_ESM.pdf]

Additional file 3. Overererexpressed proteins as indicators of astrocyte reaction

Gene name	Sum PEP Score	Abundance Ratio: (Sample) / (Control)	Abundance Ratio P-Value: (Sample) / (Control)
Gfap	330.9	4.775	2E-7
Vim	442.3	2.383	0.003
